# Genomic Fabrics of the Excretory System’s Functional Pathways Remodeled in Clear Cell Renal Cell Carcinoma

**DOI:** 10.3390/cimb45120594

**Published:** 2023-11-24

**Authors:** Dumitru Andrei Iacobas, Ehiguese Alade Obiomon, Sanda Iacobas

**Affiliations:** 1 Personalized Genomics Laboratory, Undergraduate Medical Academy, Prairie View A&M University, Prairie View, TX 77446, USA; eobiomon3@pvamu.edu; 2 Department of Pathology, New York Medical College, Valhalla, NY 10595, USA; sandaiacobas@gmail.com

**Keywords:** *ADCY6*, aldosterone-regulated sodium reabsorption, *AP2A1*, *AVP*, collecting duct acid secretion, *CREB3L4*, endocrine and other factor-regulated sodium reabsorption, *ESR1*, proximal tubule bicarbonate reclamation, vasopressin-regulated water reabsorption

## Abstract

Clear cell renal cell carcinoma (ccRCC) is the most frequent form of kidney cancer. Metastatic stages of ccRCC reduce the five-year survival rate to 15%. In this report, we analyze the ccRCC-induced remodeling of the five KEGG-constructed excretory functional pathways in a surgically removed right kidney and its metastasis in the chest wall from the perspective of the Genomic Fabric Paradigm (GFP). The GFP characterizes every single gene in each region by these independent variables: the average expression level (AVE), relative expression variability (REV), and expression correlation (COR) with each other gene. While the traditional approach is limited to only AVE analysis, the novel REV analysis identifies the genes whose correct expression level is critical for cell survival and proliferation. The COR analysis determines the real gene networks responsible for functional pathways. The analyses covered the pathways for aldosterone-regulated sodium reabsorption, collecting duct acid secretion, endocrine and other factor-regulated sodium reabsorption, proximal tubule bicarbonate reclamation, and vasopressin-regulated water reabsorption. The present study confirms the conclusion of our previously published articles on prostate and kidney cancers that even equally graded cancer nodules from the same tumor have different transcriptomic topologies. Therefore, the personalization of anti-cancer therapy should go beyond the individual, to his/her major cancer nodules.

## 1. Introduction

### Limits of the Gene Biomarker Paradigm in Cancer Diagnostics and Therapy

Cancer is a major cause of death worldwide and is likely the most funded and researched group of lethal diseases. Depending on the tumor localization, size, and metastatic stage, treatment options in specialized clinics may include surgery, chemotherapy, radiation therapy, hormone therapy, bone marrow transplantation, targeted therapy, and immunotherapy [1]. For smaller tumors, at early stages, thermal ablation offers a low-risk and minimally invasive solution [2,3]. Nevertheless, despite all the academic and industry efforts, we still do not have an efficient answer to cancer, suggesting the need for a novel approach.

According to the American Cancer Society, 52,360 men and 29,440 women are expected to be diagnosed with kidney and pelvis cancer in 2023, out of whom 9920 men and 4970 women may die from this disease [4]. The prevalence of kidney cancer is strongly dependent on age (most diagnosed people are over 65 years old), sex (twice more frequent in men than in women), and race (African Americans, American Indians, and Alaska Natives are affected in higher percentages than other races). When the cancer is localized only in the kidney, the 5-year survival rate is good (93%), however, it declines rapidly (15%) when the cancer spreads to the lungs, brain, or bones [4]. The vast majority of kidney cancers are clear cell subtypes of Renal Cell Carcinoma (ccRCC), characterized by high inter- and intra-tumor heterogeneity and strong crosstalk with the cellular microenvironment [5].

A very dynamic and promising avenue is provided by gene therapy as an alternative to kidney transplantation [6]. As of 5 October 2023, PubMed lists 143,107 articles for “cancer gene therapy” published from 1966 onward, of which 12,818 were published in 2021 alone. The majority of these articles looked for gene biomarkers whose altered sequence and expression level were supposedly responsible for triggering cancerization and whose restoration allegedly provides the cure. In most publications, the biomarkers were identified by comparing sequencing (e.g., [7,8,9] and/or transcription (e.g., [10,11,12]) data in tissues collected from cancer-stricken and healthy people.

A potentially effective yet insufficiently exploited tool for both diagnostics and therapy is quantifying and managing the amount of cancer cell-secreted microRNAs in blood and urine. Certain miRNAs have been shown to alter the expression of oncogenic or tumor-suppressive genes, thus regulating the proliferation of cancer cells. Owing to accessibility, the dosing and manipulation of the amounts of selected types of urine miRNAs was proposed as an excellent non-invasive instrument for cancer detection and management [13].

The potency of gene therapy was also tested on standard human cancer cell cultures (e.g., [14,15,16,17]), but the relevance of the experimental results from the cell culture to the cancer reality is disputable. Nonetheless, as recently summarized [18], the non-malignant cells and molecular factors from the tumor microenvironment play “crucial roles” in the development of ccRCC. Thus, when taken from their natural environment and plated in a homo-cellular culture, the cancer cells will adapt their gene expression profiles to the new conditions. Therefore, interpreting the results from homo-cellular culture as valid for the hetero-cellular tissue is disputable. We have proven that the transcriptome of one cell type changes significantly in the proximity of another cell type by profiling mouse cortical astrocytes and immortalized precursor oligodendrocytes when plated separately or co-cultured in insert systems [19].

However, what are the real predictive values of the gene biomarkers for cancer diagnosis and therapy? The 38.0 release (31 August 2023) of the NIH-National Cancer Institute GDC Data Portal [20] containing genomic data collected from 88,991 cancer cases in 68 primary sites, reported a total of 2,903,037 mutations located in 22,588 genes. Importantly, the Portal reported mutations in almost all genes affecting each of the 68 primary sites. Table 1 summarizes the GDC data for 14 primary sites by presenting the number of mutated genes found in the investigated cases, how many of the mutated genes are protein coding, and the total number of mutations detected thus far for each site.

Moreover, almost every single gene was found to be mutated in at least one case from each of the 68 primary sites. For instance, with respect to the reported cancer cases from Table 1, the titin (*TTN*) appeared to be mutated in the following percentages of reported cases: 12.41 of bladder cases, 2.75 of bone marrow, 14.47 of brain, 2.99 of breast, 5.05 of colorectal, 10.28 of head and neck, 5.71 of kidneys, 6.88 of lungs, 2.17 of pancreas, 2.60 of prostate, 13.46 of skin, 15.51 of stomach, and 12.21 of uterus cases. The percentages include all 13,073 distinct somatic mutations observed for this gene in 4512 out of the 88,991 cases included in the portal database. Thus, not only do none of the distinct mutations, but all kinds of altered sequences of *TTN* as a whole do not exhibit statistically significant sensitivity and/or specificity for a particular form of cancer. The same lack of significance is carried by all other “regular suspects”, like tumor protein p53 (*TP53*), with 1341 mutations identified in 4934 cases across 47 out of 82 projects, or *KRAS* (1500 cases, 125 distinct mutations identified in 43 projects), or *PTEN* (1228 cases, 846 mutations across 38 projects). The most frequently mutated gene in kidney cancer is the von Hippel-Lindau tumor suppressor (*VHL*) which was detected in 342 (9.77%) out of 3501 cases. However, the *VHL* was also found to be mutated in 50 cases of cancer in other organs.

An excellent recent review indicated that one possible explanation for the unsatisfactory conventional anti-cancer therapy is its targeting of the somatic tumor cells instead of the cancer stem cells (CSC), “assumed to be responsible for tumor recurrence and metastasis” [21]. Therefore, targeting the CSC-signaling pathways might offer a much better alternative than attacking the cancer-specific surface proteins.

Mimicking a human cancer phenotype in genetically engineered animals (e.g., [22,23,24,25]) provides disputable etiologies showing that together with the manipulated gene, hundreds of other genes are regulated, as reported in many studies, ours included (e.g., [26]). The set of significantly regulated genes in the tissues of genetically engineered animals, with respect to their wild-type counterparts, depends on the profiled tissue (e.g., [27]), silencing method used (e.g., [28]), and the genetic background (e.g., [29]).

Owing to the unrepeatable combination of favoring factors (some of them changing in time) of race, sex, age, medical history, diet, climate, exposure to toxins, stress, and other external stimuli, each human is a DYNAMIC UNIQUE. This dynamic unicity requires a time-sensitive personalized therapeutic approach.

Some very important factors, as of yet still neglected in many published papers and public repositories, include the tumor’s genomic, transcriptomic, and proteomic heterogeneity [30,31,32,33]. Thus, histopathologically distinct cancer nodules from the same tumor most frequently have different characteristics. Therefore, the best REFERENCE for cancer-related genomic alterations of an individual is not the tissue of the average healthy person of the same race, sex, and age group, but rather the quasi-normal tissue surrounding his/her cancer nodules [34]. With this reference in mind, the true goal of anti-cancer therapy is to restore what is considered normal for that person, hence the need for a personalized approach.

While the diagnostic value of the gene biomarkers is disputable, let us see whether the restoration of the correct sequence and/or expression level of the biomarkers can provide the therapeutic answer for cancer. Since the biomarkers are selected from the most frequently altered genes in cancer patients, it means that their sequences and/or expression levels are poorly protected by cellular homeostatic mechanisms like the minor players in cell life. Therefore, their restoration might be of little consequence. 

It is very surprising (and disappointing) that almost all gene expression studies neglect about 99.99% of the information provided by the high throughput transcriptomic platforms (RNA-sequencing, Agilent microarray, Affimetrix, Illumina BeadChip arrays, etc.), which will be presented in the Results section below. The traditional analysis considers ONLY the expression levels of the quantified genes whose comparison between conditions tells what gene was significantly up-/down-regulated (according to the arbitrarily introduced cut-off for the absolute fold change) or turned on/off. The genes are eventually clustered according to their similar behaviors across conditions (e.g., [35,36]), but similar regulation does not necessarily mean that the clustered genes are interacting with each other (they may have an upstream common regulator or transcription factor).

Using publicly available software (based on text mining the peer-reviewed literature) such as Ingenuity [37], DAVID [38], and KEGG [39], the regulated genes might be organized into functional pathways. However, the topology of the pathways constructed by such software has three major flaws: universality, rigidity, and unicity. They are universal in that they do not discriminate with respect to the strain/race, sex, age, hormonal activity, etc., and even with respect to the tissue, such as those of the Ca^2+^- and other signaling pathways. They are considered to be rigid for not changing in response to aging, medical treatment, external stimuli, and the progressions of a disease or other dynamic influencing factors. Finally, each constructed pathway has unique wiring for the genes and not a spectrum of several possible gene circuits. If two simple elements like hydrogen and carbon can combine in so many ways to form an unlimited variety of hydrocarbons, how could one assume that tens of much more complicated units (the genes) network in only a single way to accomplish a particular task? Therefore, we have used KEGG-constructed pathways only for illustrative purposes and the coordination analysis to determine the real gene networking.

Because of the above-discussed deficiencies of the biomarker approach, we switched our research from the biomarker to the Genomic Fabric Paradigm (GFP, [40]) approach. The GFP incorporates the traditional analysis of gene expression regulation while considering two additional classes of independent descriptors, and so offers the most theoretically possible comprehensive characterization of the transcriptome and personalized solutions for cancer gene therapy. The two additional transcriptomic descriptors of individual genes, the Relative Expression Variation and the Expression Correlation with each other gene, can be determined using the gene expression profiles of biological replicas without supplementary experimental costs. Through the use of the two additional groups of descriptors, the GFP approach increases by four orders of magnitude the amount of transcriptomic information extracted from a high throughput (ng RNA-sequencing or microarray) gene expression platform.

The present study complements a previously published article [40], with GFP analyses of the remodeling of the five KEGG-constructed excretion system’s functional pathways in the kidney and chest wall regions of a 74-year-old man affected by metastatic clear cell renal cell carcinoma (ccRCC).

## 2. Materials and Methods

### 2.1. The Best Choice of Tissue Samples

Nevertheless, for statistical significance, a transcriptomic study should profile several biological replicas of the compared conditions. Most authors use three biological replicas, but four is (in our view) the best compromise between getting enough statistical relevance and the errors resulting from the inherent technical noise of the profiling method. In the case of solid tumors, the most reasonable choice is to take a point biopsy from the center of a cancer nodule (or each cancer nodule, if there are more) and another one from the surrounding (almost normal) tissue, split each biopsy into four parts, and profile separately the resulted quarters. Thus, the reference for the patient’s cancer and the aim of the therapy is no longer the abstract, racially blind, ageless, and sexless model of the human body but rather his/her own normal tissue for his/her race, age, and sex. This procedure is standard in our lab and was used in investigations of surgically removed tumors from kidney [40], thyroid [41], and prostate [42] cancer patients.

In this study, we re-analyzed transcriptomic data from the surgically removed right kidney affected by ccRCC Fuhrman grade 3 (two primary cancer nodules, denoted as PTA and PTB in the renal medula) and its metastasis in the chest wall (CWM). The gene expression profiles of the three cancer nodules were compared to those of the quasi-normal surrounding kidney tissue (NOR). Data were obtained using Agilent-026652 Whole Human Genome Microarrays 4 × 44K v2 and are publicly accessible [43].

### 2.2. Data Filtering and Normalization

The hybridized microarray spots with a foreground fluorescence less than twice the background in one biological replica profiled with microarrays are eliminated from the analysis of all samples to be compared owing to the non-negligible technical noise. With this filtering, every profiled sample from all conditions to be compared was reduced to the same number of distinct transcripts, here “N = 13,314”. The background-subtracted forward fluorescence of the microarray hybridized spot(s) with transcript “*i*” from the biological replica “*k*” (*k* = 1, 2, 3, 4) of condition “c”, “*a_i_*^(*c*;*k*)^” were normalized to the expression of the median gene for that profiled sample. This normalization strategy makes comparable the expression profiles of all samples, with *a_i_*^(*c*;*k*)^ > 1 indicating genes with a higher than median expression level and *a_i_*^(*c*;*k*)^ < 1 genes with a lower than median expression level. For the analyzed microarray experiment, *a_i_*^(*c*;*k*)^ was the sum of the net fluorescence of all spots probing redundantly transcript “*i*” in the biological replica “*k*” of condition “*c*” (see Equation (A1) in Appendix A).

### 2.3. Independent Characteristics of Gene Expression

#### 2.3.1. Normalized Average Expression Level

The filtered and normalized expression values of each transcript “*i*” were averaged over the biological replicas of each condition “*c*” resulting in $AVE_{i}^{\left(c\right)}$. $AVE_{i}^{\left(c\right)}$ is the genomic measure that everybody in the field uses to determine whether that transcript abundance was up-/down-regulated or turned on/off when comparing cancer with healthy samples. Thus, the GFP includes but is not reduced to the traditional gene expression analysis.

#### 2.3.2. Relative Expression Variability

When properly selected (as quarters of point biopsies), the biological replicas may be considered as different instances of the same system subjected to distinct local (not significantly regulating) conditions. This applies to ccRCC samples owing to the strong crosstalk of cancer cells within the non-uniform microenvironment [5,18]. Thus, we can add as an independent feature of the transcript “*i*” in condition “*c*” the Relative Expression Variability, “*REV_i_^(c)^*”, computed as the mid-chi-square (*χ*^2^) interval estimate of the coefficient of variation for “*n* = 4” biological replicas and “*υ_i_*” spots probing redundantly the transcript “*i*” (Equation (A2) in Appendix A). The REV provides an indirect estimate of the strength of the cellular homeostatic mechanisms to control the transcript abundances, with the smallest REV indicating the most stably expressed (i.e., the most controlled) gene and the largest REV pointing to the most variably expressed (i.e., the least controlled) gene. Since more control means more energy spent by the cell, it is natural to assume that the right expressions of the most controlled genes were more important for the cell’s survival and/or proliferation in the multicellular tissue. As such, the REV analysis tells the investigator firsthand about the cell priorities.

#### 2.3.3. Expression Coordination

Genes are not single but team players in cell life. Considering the high efficiency of the cellular phenomena, we have introduced the Postulate of the Transcriptomic Stoichiometry (PTS) [44] as an extension to gene networking in functional pathways of Proust’s Law of Definite Proportions from chemistry [45]. The PTS states that: *in any steady-state condition, expressions of genes whose encoded products are part of a functional pathway are coordinated to ensure the maximum efficiency of that functional pathway.* This means that the involved genes are set to produce the transcripts at the right abundance proportions. The PTS and the coordination analysis can be used to determine the real gene network responsible for a particular pathway in a given condition.

The most difficult question is how the genes are networked: in pairs (e.g., agonist-antagonist), in “ménage à trois” (e.g., agonist, antagonist, and a modulator of both), or in more complex gene inter-coordination clusters? To answer this question, we adopted a formalism of correlation functions similar to that used to describe the structure of simple liquids (like liquid argon) [46]. Thus, the configuration function “*F*” of “*N*” distinct genes is considered as a superposition of virtual configurations in which the genes are: independently expressed (“*f*_1_”), coordinately expressed in pairs (“*f*_2_”), coordinately expressed in triplets (“*f*_3_”), and so on until all “*N*” genes are coordinately expressed in a cluster of all genes (“*f_N_*”). As shown in Appendix A, the contributions of the distribution functions of higher than pair-correlated genes can be neglected, so that the configuration function can be approximated with a distribution of independently expressed genes and a distribution of coordinately expressed gene pairs (Equation (A3) in Appendix A).

The problem is now to select the suitable algorithm for gene pairing. There are several weighted and unweighted types of correlation algorithms (e.g., [47,48,49,50]) aiming to identify the interconnected genes based on their co-regulation determined from the meta-analysis of genomic data on healthy and cancer-affected populations. By contrast, our aim is to determine the gene network in a particular condition (normal or cancerous) of only one individual, so none of these cluster analyses are suitable for our endeavor.

The simplest gene pairing is completed using the Pearson pair-wise correlation coefficient (hereafter denoted as “*COR*” instead of the traditional “*r*”) between the log_2_ of the normalized expressions of two genes (“*i*” and “*j*”) in the four replicas of the same condition “*c*” (shown in Equation (A4) in Appendix A). Although Marbach et al. [51] have shown that Pearson’s correlation coefficient is not the strongest way to determine gene networks, it is accurate enough when taking into account the technical noise of the gene expression platform. With four biological replicas, two genes are (*p* < 0.05) significantly synergistically expressed (i.e., a positive correlation) if *COR* ≥ 0.951, antagonistically expressed (negative correlation) if *COR* ≤ −0.95, and independently expressed (null correlation) if |*COR*| ≤ 0.05. For microarrays probing the same transcript with two spots (i.e., 8 paired values), the *p* < 0.05 significance cut-off for synergism/antagonism is |*COR*| ≥ 0.71, for three spots (12 paired values) it is |*COR*| ≥ 0.58, and so on, with the Pearson cut-off decreasing when the number of probing spots increases [52].

In [53], we defined the coordination score “*COORD*” with *p*-value “*p*” of a pathway “*Γ*” in condition “*c* = *NOR*, *PTA*, *PTB*, *CWM*” as:

(1) $${COORD}_{\Gamma }^{\left(c\right)}\left(p\right)\equiv {SYN}_{\Gamma }^{\left(c\right)}+{ANT}_{\Gamma }^{\left(c\right)}-{IND}_{\Gamma }^{\left(c\right)}$$

where “*SYN*/*ANT*/*IND*” are the percentages of all gene pairs from the pathway “*Γ*” that are synergistically/antagonistically/independently expressed with a statistical significance (*p*-value) “*p*” in condition “*c*”.

The *COORD* score indicates the (*p* > 0.05) statistically significant influence of that gene on all other genes.

#### 2.3.4. Topology of the Transcriptome and the Gene Master Regulator

The “*REV*” and “*COR*” can be used to determine the Gene Commanding Height (*GCH*) that establishes the importance hierarchy of the genes in each region. The top gene (highest *GCH*) is termed the Gene Master Regulator (GMR) of that region. The GMR is the highly protected gene (i.e., low *REV*, meaning it is critical for cell survival) that also has the strongest influence on the expression of other genes through expression coordination [54,55] (Equation (A5) in Appendix A). In all our cancer genomics studies to date ([34,40,41,42,54,55]), we found that cancer and normal cells from the same tumor are controlled by different GMRs. Moreover, the GMR gene of the cancer cells has low GCH in the normal cells. Therefore, silencing the GMR of the cancer cells is expected to selectively kill the cancer cells from the tissue with very little influence on the normal cells.

Note: For the GFP users, the transcriptome is no longer a chaotic collection of transcripts, but a multi-dimensional hierarchized mathematical entity subjected to dynamic sets of expression variability and expression correlations of its components.

### 2.4. Transcriptome Alteration in Cancer

#### 2.4.1. Measures of Expression Regulation

We use the GFP to compare the transcriptome of a cancer nodule with that of the surrounding normal tissue in a tumor. Thus, in addition to identifying what genes were significantly up-/down regulated in cancer, we determine also how much of the homeostatic control of the transcript abundance was altered for each gene or group of genes, and how the gene networks were remodeled.

The traditional analysis considers a gene as significantly regulated in cancerous tissue compared to normal tissue if the expression ratio “*x*” (negative for down-regulation) has an absolute fold-change |*x*| larger than an arbitrarily introduced cut-off (most frequently 1.5). In most cases, it also adds the condition that the *p*-value of the heteroscedastic *t*-test of mean expressions is less than 0.05. However, the cut-off fold-change could be too stringent for very stably expressed genes (leading to false negatives) or too lax for very unstably expressed ones (introducing false positives). Therefore, we use for each gene the cut-off of the absolute fold-change, “*CUT*”, considering the combined contributions of its biological variability and the technical noises of the platform in the two profiled conditions. Thus, the gene “*i*” is considered significantly regulated in “cancer” with respect to the normal tissue (*NOR*), if the absolute fold-change exceeds the respective “*CUT*” and the *p*-value is less than 0.05 (Equation (A6) in Appendix A).

In many studies, transcriptome alteration is presented as percentages of the significantly up- and down-regulated genes. Nonetheless, the percentage of presentation implicitly assumes that all regulated genes are uniform (+1 or −1) contributors to the overall transcriptome alteration while neglecting the contributions of the not significantly regulated genes. A more accurate measure of the expression regulation is the Weighted Individual (gene) Regulation “*WIR*”, whose absolute values can be averaged for the genes included in a functional pathway “*Γ*” as the Weighted Pathway Regulation “*WPR*”. In addition to being applied to all genes (including those not significantly regulated), *WIR* takes also into account the absolute expression change and the statistical significance of the regulation (as shown in Equations (A7) and (A8) in Appendix A).

#### 2.4.2. Regulation of the Control of Transcript Abundance

**Figure 1:**
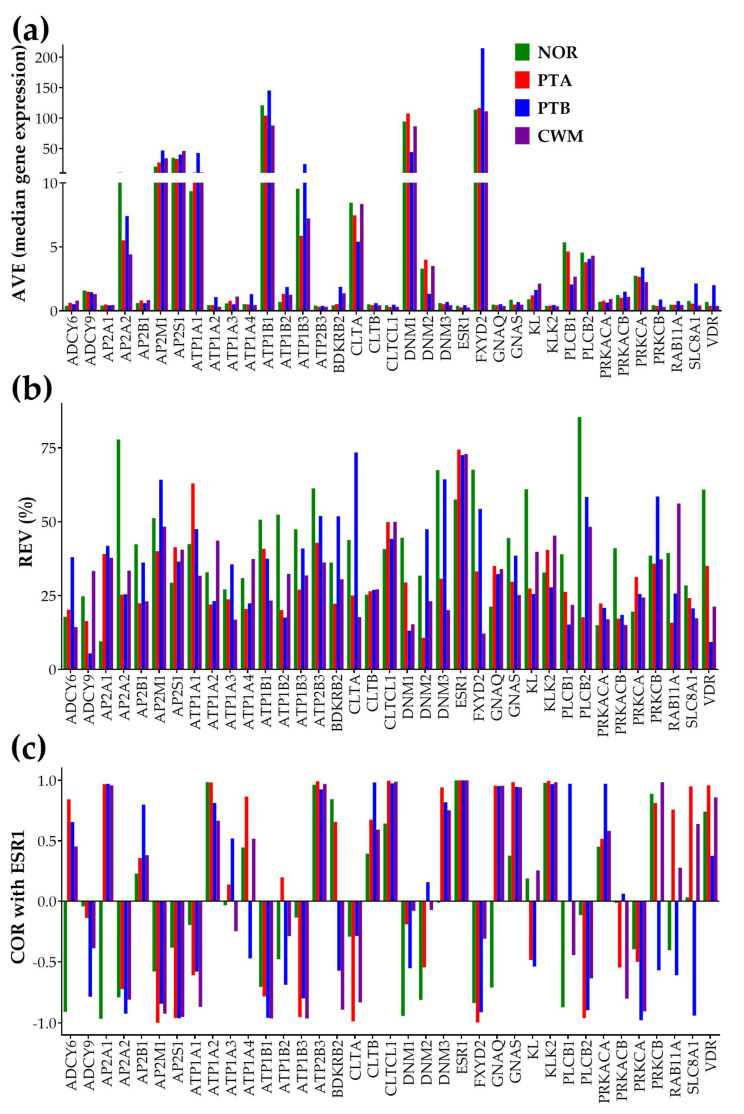
Independence of: (**a**) AVE, (**b**) REV, and (**c**) COR with *ESR1* characteristics for 37 genes involved in the KEGG-constructed pathway of the “Endocrine and other factor-regulated calcium absorption”. The COR_ESR1,ESR1_ = 1 values in all conditions validate the coordination analysis. Observe the differences in all three gene characteristics between the two equally graded and located close to each other nodules of PTA and PTB.

**Figure 2:**
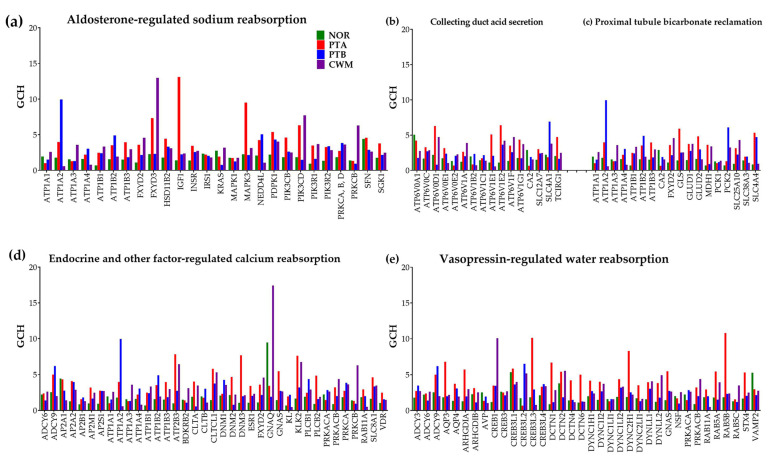
Gene Commanding Height (GCH) scores of the genes involved in the five KEGG-constructed excretory pathways: (**a**) Aldosterone-regulated sodium reabsorption, (**b**) Collecting duct acid secretion, (**c**) Proximal tubule bicarbonate reclamation, (**d**) Endocrine and other factor-regulated calcium reabsorption, and (**e**) Vasopressin-regulated water reabsorption.

In a previous paper [56], we defined the Relative Expression Control, “*REC*,” of gene “*i*” in condition (here region) “*c*” so that positive *REC*s indicate more than the median-controlled genes and negative values indicate less than the median-controlled genes in that condition (shown as Equation (A9) in Appendix A). As shown below (Figure 1), the ccRCC altered the *REV*s of individual genes and as a consequence, their hierarchy (illustrated in Figure 2 below). The difference between the REV inverses in a cancer nodule and those in the surrounding normal tissue (Equation (A10) in Appendix A) indicates how much the ccRCC altered the transcript abundance control.

#### 2.4.3. Regulation of Expression Coordination

The regulation of expression coordination can be computed with regard to a single gene, a group of genes (like those involved in a particular pathway), or all quantified genes. In this report, the regulation of the expression coordination is limited to the five KEGG-constructed excretory system pathways (shown in Equation (A11) in Appendix A). Positive regulation values indicate an overall increase in the expression coordination while negative values indicate an overall decrease in the expression coordination of gene “*i*” with expressions of all genes from the reference pathway “*Γ*”.

#### 2.4.4. The Transcriptomic Distance

Nonetheless, the most comprehensive measure of the transcriptome alteration should include all changes: in expression level, expression control, and expression coordination with all other genes. The Transcriptomic Distance of an Individual gene, “*TDI*”, is the Euclidian distance from the origin of the 3D space whose orthogonal axes are: WIR, the regulation of the expression control, and the regulation of expression coordination (shown in Equation (A12) in Appendix A).

### 2.5. Functional Pathways

In this report, we used our experimental data from the profiled samples of a man with metastatic ccRCC [43] to determine the topology and the ccRCC-induced remodeling of the transcriptomes associated with the five KEGG-constructed functional pathways of the excretory system. The analyzed pathways were: (ALDO) hsa04960 Aldosterone-regulated sodium reabsorption [57], (COLL) hsa04966 Collecting duct acid secretion [58], (ENDO) hsa04961 Endocrine and other factor-regulated calcium reabsorption [59], (PROX) hsa04964 Proximal tubule bicarbonate reclamation [60], and (VASO) hsa04962 Vasopressin-regulated water reabsorption [61].

## 3. Results

### 3.1. The Global Picture

Expressions of 13,314 unigenes were adequately quantified, normalized, and organized in functional pathways in all 16 samples. However, not all excretory genes identified by the KEGG were analyzed. The missing genes were the ones that: (i) were not expressed in one of the four profiled regions, (ii) had no microarray spot to be probed by, or (iii) were probed by spots excluded from the analysis because of corrupted pixels.

Nonetheless, the functional pathways of the excretory system were not mutually exclusive, some genes being counted in two (e.g., *ADCY9* in ENDO and VASO) or even three excretory pathways (e.g., *ATP1A1* in ALDO, ENDO, and PROX). Therefore, the total number of the quantified distinct excretory genes was 108.

### 3.2. Independent Characteristics of Gene Expression

Figure 1 illustrates the independence of the three types of expression characteristics for 37 genes involved in the KEGG-constructed pathway of the “Endocrine and other factor—regulated calcium reabsorption” [59] using our microarray data on Fuhrman grade 3 metastatic ccRCC samples [38]. For the COR analysis, we chose to represent the expression correlation with the estrogen receptor 1 (*ESR1*), owing to the kidney being one of “the most estrogen-responsive, not reproductive organs in the body” [62].

Of note are the differences between the normal tissue and the cancer nodules not only in the average expression levels of certain genes (AVE, as expected and reported by the traditional analysis), but also in the relative expression variability (REV) and correlation (COR) with other genes (here with *ESR1*). For instance, the significant expression antagonism (COR^(NOR)^ = −0.96624, *p* = 0.0338) of *AP2A1* (adaptor-related protein complex 2, alpha 1 subunit) with *ESR1* in the normal tissue is switched into expression synergism in each of the three cancer nodules. We obtained the following correlation values (and statistical significances) between *ESR1* and *AP2A1* in the cancer samples: COR^(PTA)^ = 0.9607 (*p*-val = 0.03093), COR^(PTB)^ = 0.970449 (*p*-val = 0.02955), and COR^(CWM)^ = 0.958562 (*p*-val = 0.04144). This result means that in the normal tissue, when the expression of *ESR1* goes up, that of *AP2A1* goes down, and when *ESR1* goes down, *AP2A1* goes up. By contrast, in cancer, the expression of *ESR1* oscillates in-phase with the expression of *AP2A1*, so that expressions of both genes go up or down simultaneously. It is interesting to also note the differences in all three characteristics of the individual genes between the nodules of PTA and PTB.

The independence of the three characteristics of the individual genes collected from a regular gene expression experiment with four biological replicas of each histopathologically distinct region is visually evident. Therefore, each of the three types of analyses brings non-redundant information and is worth taking into account.

### 3.3. ccRCC Changed the Gene Hierarchy

Figure 2 presents the GCH scores for all quantified genes selected by the KEGG as involved in the five excretory functional pathways. Of note are again the substantial inter-regional differences in the genes’ GCH scores, indicating distinct gene hierarchies within the corresponding pathways. For instance, the GCH of the *IGF1* (insulin-like growth factor 1 (somatomedin C)) from the “Aldosterone-regulated sodium reabsorption” pathway increased from 1.41 in NOR to 13.11 (9.28×) in PTA, although it remains practically unchanged in PTB (2.25) and in CWM (2.38). The *CREB3L2* (cAMP responsive element binding protein 3-like 2) from the “Vasopressin-regulated water reabsorption” pathway exhibited a GCH increase of 9.57× in PTB compared to PTA. The GCH of the *ATP1A2* (ATPase, Na^+^/K^+^ transporting, alpha 2 polypeptide) decreased by 16.85× in CWM with respect to PTB.

### 3.4. Measures of Individual Gene Regulation

**Figure 3:**
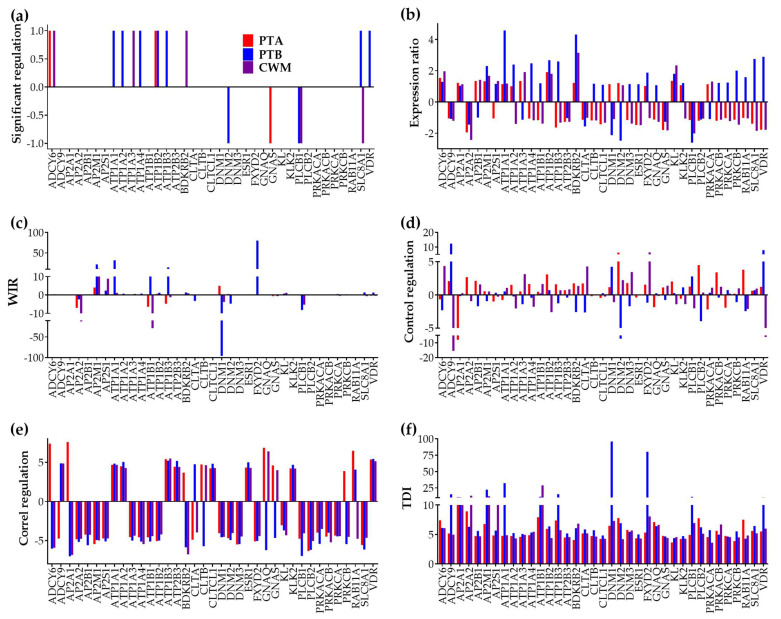
Six measures of individual gene regulation in the KEGG-constructed pathway of ENDO (Endocrine and other factor-regulated calcium reabsorption). (**a**) Uniform (+1/−1 for significant up-/down regulation). (**b**) Expression ratio × (negative for down-regulation). (**c**) Weighted individual (gene) regulation (WIR, negative for down-regulation). (**d**) Regulation of transcript abundance control mechanisms (negative for decreased control). (**e**) Regulation of expression coordination (with respect to every other gene of the pathway, negative for reduced correlation); (**f**) Transcriptomic distance of individual (gene) (here with respect to all its partners within the pathway*).* Observe that all but the uniform measure takes into account the contributions of every single gene.

Figure 3 illustrates the six types of quantifying measures of the ccRCC-induced alterations of the genes from the KEGG-constructed pathway hsa04961 of “Endocrine and other factor-regulated calcium reabsorption”. The six measure types are: the uniform +1/−1 for significant regulation, the expression ratio (negative for the down-regulation), the WIR (Weighted Individual (gene) Regulation, negative for down-regulation), the regulation of the transcription control, the regulation of the expression correlation with all other genes from the pathway, and the TDI (Transcriptomic Distance of Individual gene).

There are notable differences among the three cancer nodules in all six measures. For instance, *SLC8A1* (solute carrier family 8 (sodium/calcium exchanger), member 1), which was not regulated in PTA, was significantly up-regulated in PTB (x = 2.75) and significantly down-regulated in CWM (x = −1.84). Only one gene, *ATP1B2* (ATPase, Na^+^/K^+^ transporting, beta 2 polypeptide), was significantly upregulated in both PTA (x = 1.91) and PTB (x = 2.67), but not in CWM. *PLCB1* (phospholipase C, beta 1 (phosphoinositide-specific) was the single gene significantly down-regulated in PTB (x = −2.60) and CWM (x = −2.01), but not in PTA.

As defined, the WIR takes larger absolute values for highly expressed genes in the reference (here NOR) region, sometimes even larger for not significantly regulated genes than for significantly regulated ones. For instance, *DNM2* (dynamin2), which is a significantly down-regulated gene in PTB (x^(PTB→NOR)^ = −2.47, CUT^(PTB→NOR)^ = 1.81, *p*^(PTB→NOR)^ = 0.0242), had a WIR^(PTB→NOR)^ = 4.73. The *DNM2* contribution to the transcriptomic alteration in PTB was substantially overpassed by that of the not statistically significantly regulated *DNM1* (dynamin1: WIR^(PTB→NOR)^ = 95.74, x^(PTB→NOR)^ = −2.12, CUT ^(PTB→NOR)^ = 1.66, *p*^(PTB→NOR)^ = 0.0969 > 0.05). The reason for this is that in NOR, *AVE_DNN1_* = 94.30 >> 3.30 = *AVE_DNN2_.* Nonetheless, by considering the whole change in the expression level, the WIR tells the investigator much more about the contribution of a gene to the transcriptome expression alteration than the uniform +1/−1 up-/down regulation.

Overall, the median expression control 100/<REV> increased from 2.58 in NOR to 4.27 in PTA, 2.92 in PTB, and 3.96 in CWM. In the illustrated pathway, we found genes with a substantial increase in expression control and genes with a substantial decrease in control in cancer. The most substantial increase of the expression control was for *ADCY9* (adenylate cyclase 9) in PTB (Δ*REC*^(*PTB*→*NOR*)^ = 12.38). It is remarkable that the control of *ADCY9* had a modest change in PTA (Δ*REC*^(*PTA*→*NOR*)^ = 2.08) but the largest decrease of all in CWM (Δ*REC*^(*CWM*→*NOR*)^ = −15.50). The largest decrease in PTA was exhibited by *AP2A1* (adaptor-related protein complex 2, alpha 1 subunit) with Δ*REC*^(*PTA*→*NOR*)^ = −7.97, but with insignificant changes in the other nodules with Δ*REC*^(*PTB*→*NOR*)^ = −0.17, and Δ*REC*^(*CWM*→*NOR*)^ = 0.26. Interestingly, there are genes (e.g., *DNM2*) whose control increased with respect to NOR in one cancer nodule (PTA, Δ*REC*^(*PTA*→*NOR*)^ = 6.2) but decreased in the equally ranked other nodule from the same tumor (Δ*REC*^(*PTB*→*NOR*)^ = −7.2), indicating a shift in the cell’s priorities. A substantial shift in cell priorities occurred also for *VDR* (vitamin D (1,25-dihydroxyvitamin D3) receptor) between the nodules of PTB (Δ*REC*^(*PTB*→*NOR*)^ = 7.92) and CWM (Δ*REC*^(*CWM*→*NOR*)^ = −6.07).

We found genes, such as *ADCY6*, with an increased overall correlation with the other pathways’ genes with respect to NOR in one nodule (Δ*COR*^(*PTA*→*NOR*)^ = 7.39) but decreased in the other nodules (Δ*COR*^(*PTB*→*NOR*)^ = −6.04, Δ*COR*^(*PTB*→*NOR*)^ = −5.92), indicating a profound remodeling of the gene networking. Interestingly, *ADCY6* was significantly up-regulated in PTA (x = 1.54) and CWM (x = 1.96), but not in PTB (x = 1.29). A very similar behavior, except that it was not significantly regulated in any of the three cancer nodules, was exhibited by *AP2A1* (Δ*COR*^(*PTA*→*NOR*)^ = 7.60, Δ*COR*^(*PTB*→*NOR*)^ = −7.02, Δ*COR*^(*PTB*→*NOR*)^ = −6.84).

Nevertheless, the most comprehensive measure that incorporates the changes in all three types of characteristics is the transcriptomic distance of an individual gene (TDI) from its AVE^(*NOR*)^, REV^(*NOR*),^ and COR^(*NOR*)^ (with all other genes within the pathway) in the normal tissue. From the TDI perspective, *DNM1* (TDI^(*PTB*→*NOR*)^ = 95.95 in PTB) followed by *FXYD2* (TDI^(*PTB*→*NOR*)^ = 80.21 in PTB) were the most altered genes within this set. Interestingly, with all differences at the individual gene level, the median TDIs of the three nodules were close to each other (5.72 for PTA, 5.73 for PTB, and 5.37 for CWM).

### 3.5. Overall Regulation of the Excretory Pathways

Table 2 presents the overall gene expression alterations of the five KEGG-constructed excretory pathways as percentages of up- and down-regulation out of the quantified genes in each of the cancer nodules. Table 2 presents also the WPRs of the analyzed pathways.

With 23.08% in PTA and CWM, and 30.77% in PTB total percentage of up- and down-regulated genes, the ALDO appears as the most altered pathway. However, from the more comprehensive WPR perspective, the COLL is the most altered of the five pathways in all three cancer nodules.

Interestingly, all significantly regulated genes are up for PROX in all three cancer nodules, indicating a major activation of this pathway in ccRCC. The results on the ALDO are intriguing: while there were equal numbers of up- and down-regulated genes (11.54% of 26) in both PTA and CWM, in PTB all 30.77% significantly regulated genes were over-expressed. This means that the ALDO was balanced in PTA and CWM, but strongly activated in PTB. The ALDO regulomes (sets of significantly regulated genes in this pathway) of the three cancer nodules are different, with only one gene, *PIK3R2* (phosphoinositide-3-kinase, regulatory subunit 2 (beta)), being significantly up-regulated in all three nodules.

Results from Table 2 show that even when closely located and with equal pathology grades, cancer nodules from the same tumor (PTA and PTB) may exhibit different gene alterations, questioning the validity of meta-analyses comparing ccRCC patients with healthy counterparts.

### 3.6. False Hits

**Figure 4:**
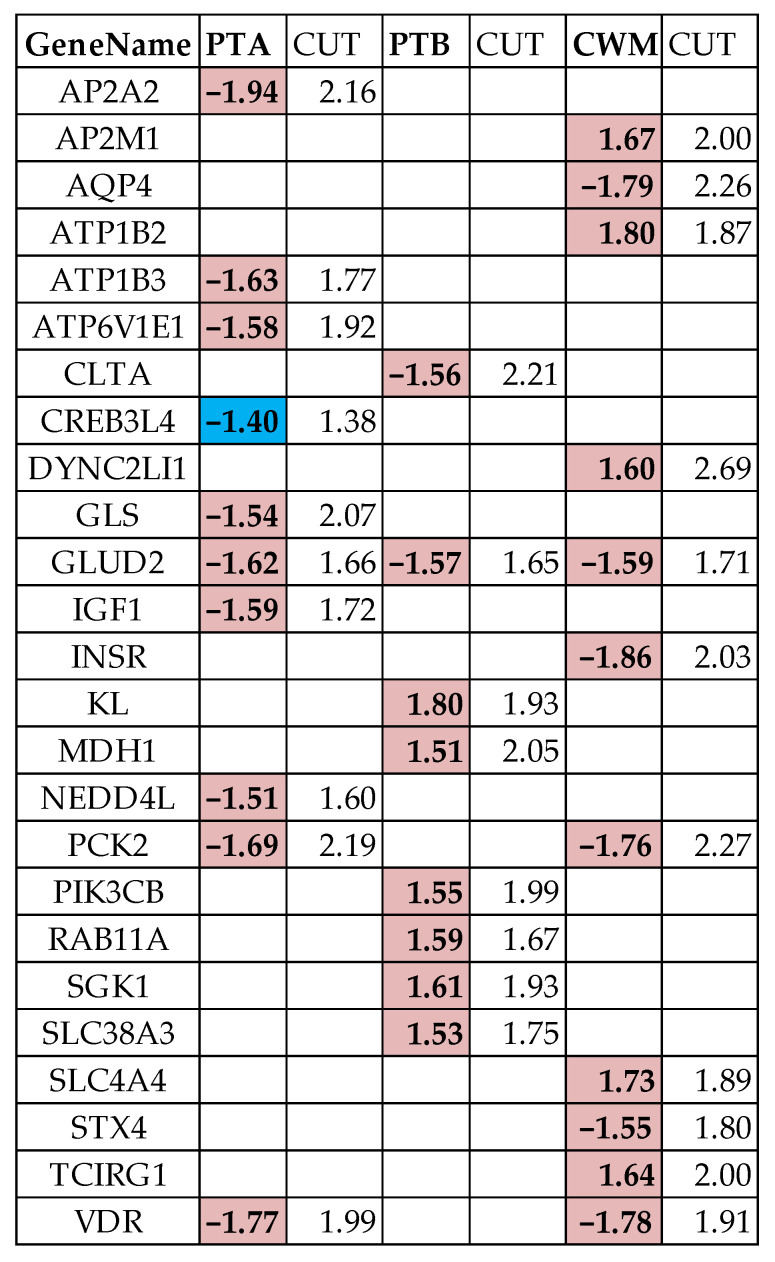
Excretory genes identified by our absolute fold-change criterion (|x^(cancer→NOR)^| > CUT^(cancer→NOR)^) as false positive hits (red accent 2 lighter 60% background) and false negative hits (light blue background) in the traditional analysis of gene expression. PTA/PTB/CWM = expression ratio (negative for down-regulation) in the indicated cancer nodule with respect to the normal tissue (NOR). CUT = absolute fold-change cut-off to consider a gene as significantly regulated.

Figure 4 presents the excretory genes that would have been considered as significantly regulated in the traditional analysis (|x^(cancer→NOR)^| > 1.5) but were identified by our cut-off criterion as false positive hits (|x^(cancer→NOR)^| ≤ CUT^(cancer→NOR)^). In contrast, the *CREB3L4* (cAMP responsive element binding protein 3-like 4) was identified as a false negative hit (|x^(PTA→NOR)^| < 1.50) in PTA because |x^(PTA→NOR)^| = 1.40 > CUT^(PTA→NOR)^ = 1.38). In consequence, the false positive hits were eliminated, and the false negative hits were included in the “excretory regulomes” of the three cancer nodules.

### 3.7. Location of the Regulated Genes in the Excretory System’s Functional Pathways

**Figure 5:**
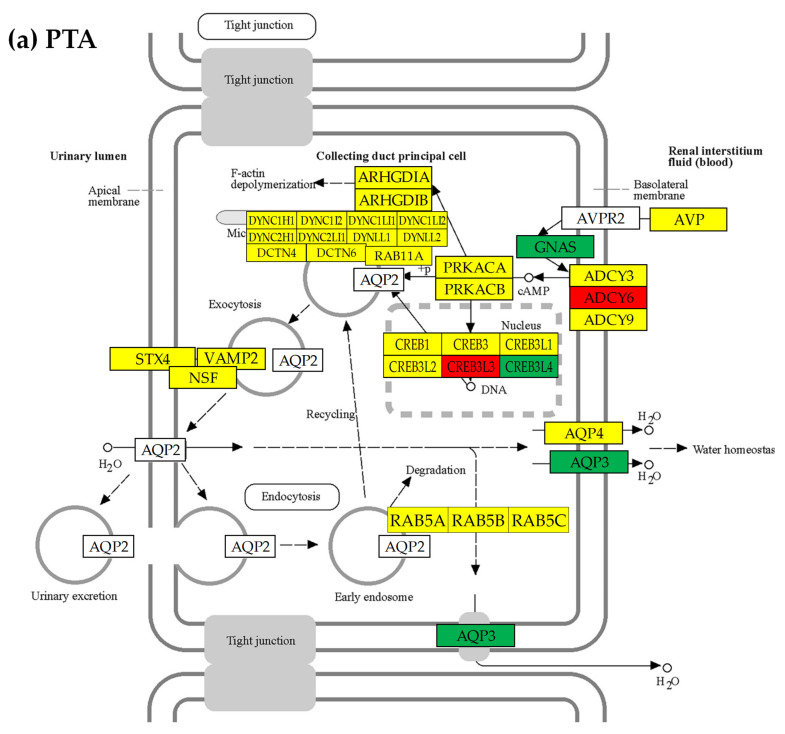

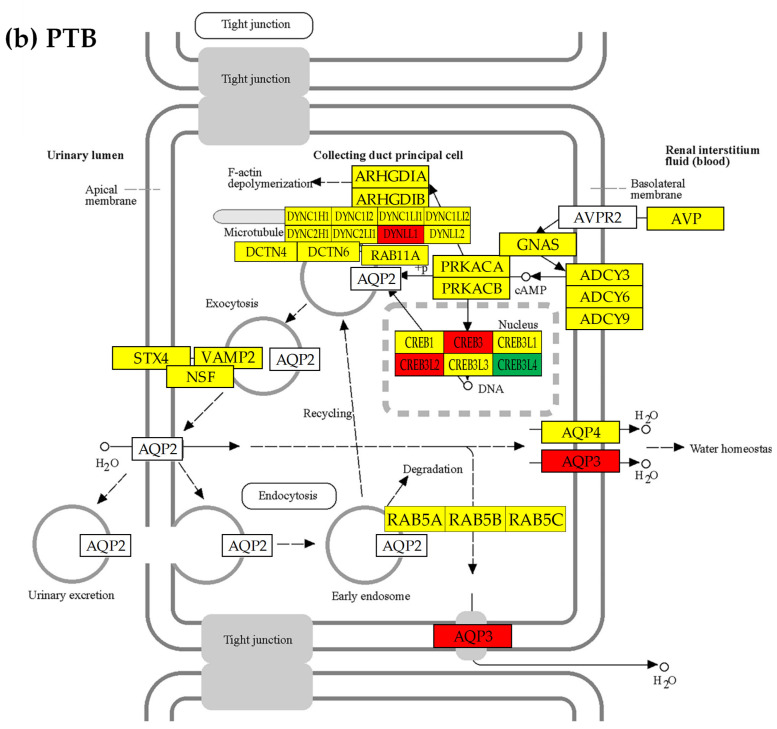

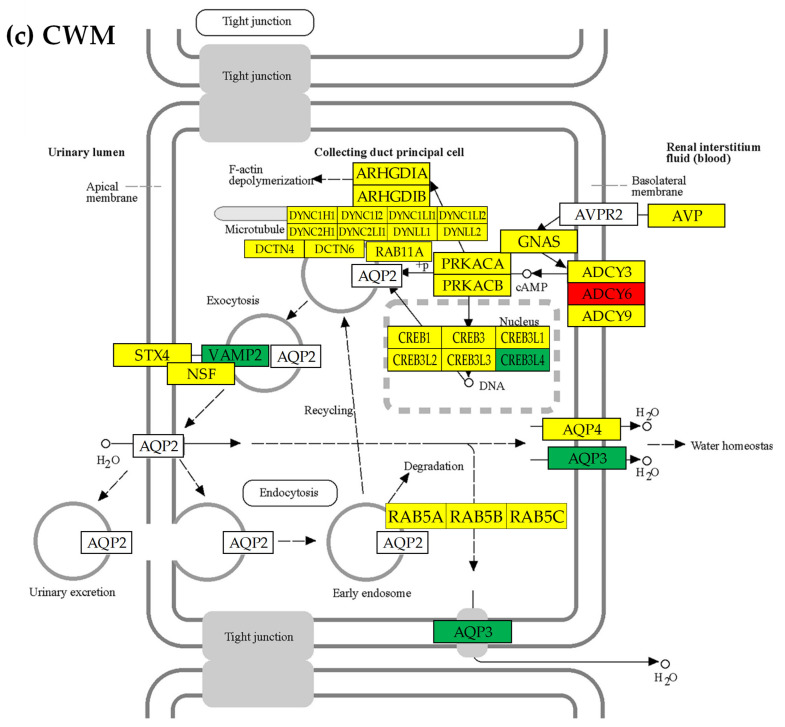
The regulated genes from the KEGG-constructed pathway of hsa04962 “Vasopressin-regulated water reabsorption” in the three cancer nodules with respect to the surrounding normal (NOR) tissue in the right kidney: (**a**) PTA, (**b**) PTB, and (**c**) CWM. Red/green background of the gene symbol indicates significant up-/down-regulation, yellow background indicates not statistically significant regulation, while blank background indicates that that gene was not quantified. Significantly regulated genes: *ADCY6* (adenylate cyclase 6), *AQP3*, *CREB3* (cAMP responsive element binding protein 3), *CREB3L2*/*3*/*4* (cAMP responsive element binding protein 3-like 2/3/4), *DCTN1*/*2* (dynactin 1/2), *DYNC2LI1* (dynein, cytoplasmic 2, light intermediate chain 1), *GNAS* (GNAS complex locus), and *VAMP2* (vesicle-associated membrane protein 2 (synaptobrevin 2)). Note the differences among the three nodules including that *AQP3* is down-regulated in PTA and CWM, but up-regulated in PTB.

See Figure 5 for the hsa04962 (VASO) “Vasopressin-regulated water reabsorption” and Figures S1–S4 from Supplementary Material for the other four KEGG-constructed excretory system pathways present for every profiled cancer nodule and the localizations of the regulated genes. Of note are the inter-nodule differences in the subsets of the regulated genes.

We found that although the VASO gene of *AVP* (arginine vasopressin) was not significantly regulated in any of the three cancer nodules, expressions of several other genes were significantly altered (though not in the same way) in all profiled regions. For instance, the *AQP3* (aquaporin 3 (Gill blood group)) was found as down-regulated in PTA (x^(PTA→NOR)^ = −2.808) and CWM (x^(CWM→NOR)^ = −5.846) but up-regulated in PTB (x^(PTB→NOR)^ = 2.034).

The opposite regulations of the *AQP3* in the two closely located and equally pathologically graded nodules of PTA and PTB is another argument to consider the “transcriptomic signature” unreliable for ccRCC [63]. The GDC data Portal of the National Cancer Institute reports four cases of kidney cancer (three females and one male) where *AQP3* was found to be mutated.

Unfortunately, the important *AQP2* (aquaporin 2) and *AVPR2* (arginine vasopressin receptor 2) were not quantified in this experiment.

Only two excretory genes were similarly regulated in all three cancer nodules. The VASO gene *CREB3L4* (cAMP responsive element binding protein 3-like 4) was down-regulated: x^(PTA→NOR)^ = −1.40 (CUT ^(PTA→NOR)^ = 1.38, *p*^(PTA→NOR)^ = 0.040); x^(PTB→NOR)^ = −1.74 (CUT^(PTB→NOR)^ = 1.63, *p*^(PTB→NOR)^ = 0.031), x^(CWM→NOR)^ = −1.95 (CUT^(CWM→NOR)^ = 1.48, *p*^(CWM→NOR)^ = 0.003). The down-regulation of the *CREB3L4* gene in PTA would have been considered not significant in the traditional analysis requiring |x^(PTA→NOR)^| > 1.50, but was identified as significant by our algorithm that requires the absolute fold-change to exceed the cut-off value computed for that gene in the compared samples.

In contrast, the ALDO gene *PIK3R2* (phosphoinositide-3-kinase, regulatory subunit 2 (beta)) was upregulated in all three cancer nodules: x^(PTA→NOR)^ = 3.31 (CUT^(PTA→NOR)^ = 1.89, *p*^(PTA→NOR)^ = 0.013), x^(PTB→NOR)^ = 1.85 (CUT^(PTB→NOR)^ = 1.82, *p*^(PTB→NOR)^ = 0.046), x^(CWM→NOR)^ = 3.30 (CUT^(CWM→NOR)^ = 1.90, *p*^(CWM→NOR)^ = 0.032).

Two genes were oppositely regulated in the nodules of PTB and CWM: the ALDO gene *SFN* (stratifin; x^(PTB→NOR)^ = 2.03, x^(CWM→NOR)^ = −1.95) and the ENDO gene *SLC8A1* (solute carrier family 8 (sodium/calcium exchanger), member 1; x^(PTB→NOR)^ = 2.75, x^(CWM→NOR)^ = −1.84).

Three genes were similarly regulated in PTA and PTB: the *ATP1B2* (ATPase, Na^+^/K^+^ transporting, beta 2 polypeptide; x^(PTA→NOR)^ = 1.91, x^(PTB→NOR)^ = 2.67), the *DCTN2* (dynactin 2 (p50); x^(PTA→NOR)^ = −1.51, x^(PTB→NOR)^ = −2.06), and the *SLC4A4* (solute carrier family 4 (sodium bicarbonate cotransporter), member 4; x^(PTA→NOR)^ = 2.19, x^(PTB→NOR)^ = 2.57).

Three regulated genes in PTA were similarly regulated in CWM: the *ADCY6* (adenylate cyclase 6; x^(PTA→NOR)^ = 1.52, x^(CWM→NOR)^ = 1.96), the *KRAS* (Kirsten rat sarcoma viral oncogene homolog; x^(PTA→NOR)^ = 2.21, x^(CWM→NOR)^ = 2.77), and the *PIK3CD* (phosphatidylinositol-4,5-bisphosphate 3-kinase, catalytic subunit delta; x^(PTA→NOR)^ = −1.65, x^(CWM→NOR)^ = −1.97).

Two genes were similarly regulated in PTB and CWM: the *CA2* (carbonic anhydrase II; x^(PTB→NOR)^ = 9.94, x^(CWM→NOR)^ = 3.44) and the *PLCB1* (phospholipase C, beta 1 (phosphoinositide-specific), x^(PTB)^ = −2.60, x^(CWM)^ = −2.01).

### 3.8. Tumor Heterogeneity of the Transcriptomic Networks

**Figure 6:**
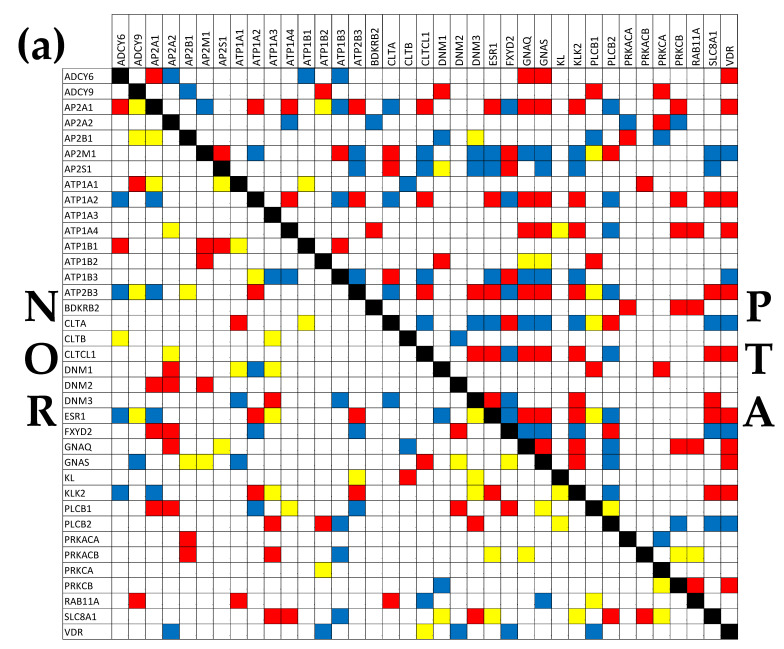

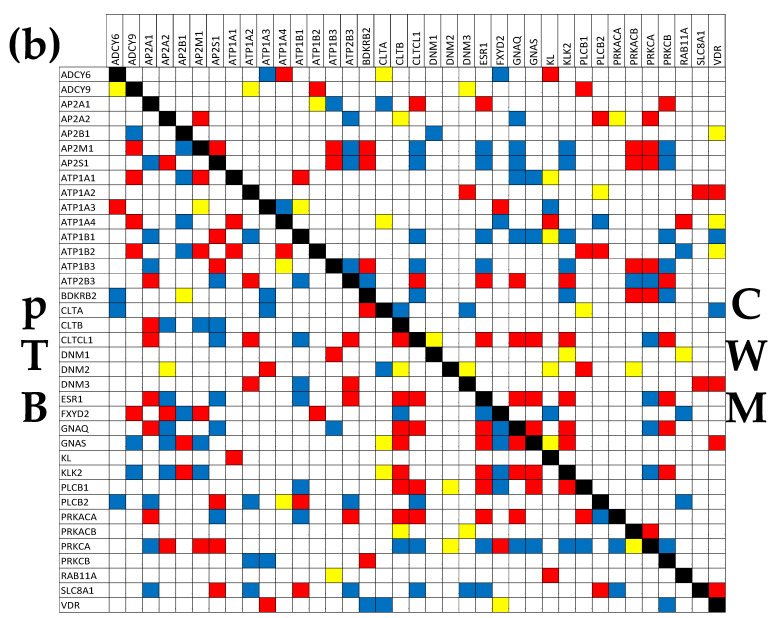
(*p* < 0.05) significant synergism, antagonism, and independence among the genes responsible for the Endocrine and other factor-regulated calcium reabsorption. (**a**) Significant gene expression correlations in NOR and PTA. (**b**) Significant gene expression correlations in PTB and CWM. A red/blue/yellow square indicates significant synergism/antagonism/independence of the genes labeling the intersecting row and column, while a blank square means a lack of statistical significance of the expression correlation.

Figure 6 presents the (*p* < 0.05) significant synergism, antagonism, and independence among the genes from the hsa04961 pathway of ENDO (Endocrine and other factor-regulated calcium reabsorption) [59]. It is interesting to observe that the percentage of the synergistic pairs increased from 12.28% in NOR to 26.90% in PTA, 20.76% in PTB, and 16.96% in CWM. The percentage of the antagonistic pairs increased from 9.65% in NOR to 21.92% in PTA, 20.76% in PTB, and 16.08% in CWM, while that of the independently expressed pairs decreased from 12.28% in NOR to 4.09% in PTA, 4.68% in PTB, and 6.43% in CWM. Altogether, the coordination score COORD = %synergistic + %antagonistic − %independent increased from 9.65% in NOR to 44.74% in PTA, 36.84% in PTB, and 26.61% in CWM. These results indicate a substantial ccRCC-triggered increase in the inter-coordination of the genes involved in this pathway.

Of note are again the substantial differences between the PTA and PTB regions, indicating distinct wiring of the genes in the functional network. For instance, there are 7 genes (*AP2S1*, *ATP1A2*, *ATP2B3*, *CLTCL1*, *DNM3*, *ESR1*, and *PLCB2*) whose significant correlations with the sodium/calcium exchanger *SLC8A1* are opposite in the two kidney nodules, indicating major differences in the gene networking. Thus, the *ATP1A2*, *ATP2B3*, *CLTCL1*, *DNM3*, and *ESR1* are synergistically expressed with the *SLC8A1* in PTA but antagonistically expressed with the *SLC8A1* in PTB, while the *AP2S1* and *PLCB2* are antagonistically expressed with the *SLC8A1* in PTA but synergistically expressed with the *SLC8A1* in PTB.

**Figure 7:**
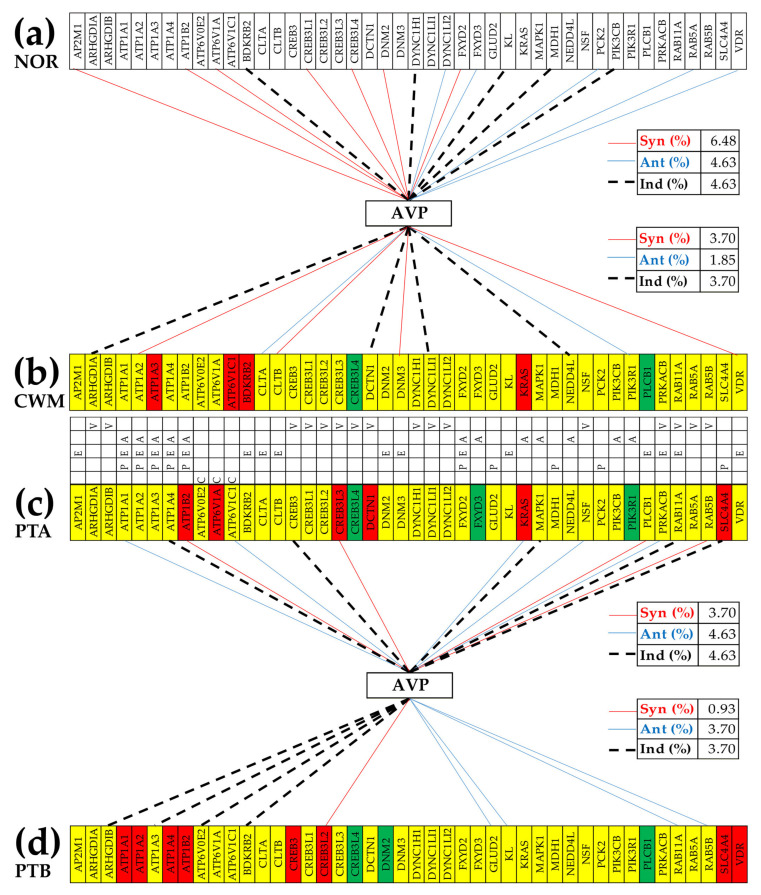
Statistically significant synergism, antagonism, and independence of excretory genes with arginine vasopressin (*AVP*) in all four profiled regions. (**a**) Significant expression correlation partners of *AVP* in NOR. (**b**) Significant expression correlation partners of *AVP* in CWM. (**c**) Significant expression correlation partners of *AVP* in PTA. (**d**) Significant expression correlation partners of *AVP* in PTB. A continuous red/blue line indicates synergism/antagonism, while a dashed black line indicates significant independence. Letters A, C, E, P, and V indicate the pathway affiliations of the genes: A = Aldosterone-regulated sodium reabsorption, C = Collecting duct acid secretion, E = Endocrine and other factor-regulated calcium reabsorption, P = Proximal tubule bicarbonate reclamation, and V = Vasopressin-regulated water reabsorption. Note: only the genes with significant correlations in at least one region were included in the figure.

Figure 7 presents the (*p* < 0.05) statistically significant synergism, antagonism, and independence of the quantified excretory genes with *AVP* in all four regions profiled. In the KEGG-constructed “Vasopressin-regulated water reabsorption” (VASO) pathway, *AVP* is directly connected to *AVPR2* (arginine vasopressin receptor 2; not quantified in the experiment) and indirectly connected to *GNAS.* Using the COR analysis, we found that in the normal kidney *AVP* is significantly connected to the *CREB3L1*, *CREB3L4*, *DYNC1H1*, and *RAB5A*.

The cancer reconfigures the VASO networks, so that in PTA, the *AVP* is significantly connected to the *CREB3L3*, *NSF*, *PRKACB*, and *RAB5B.* In PTB, the *AVP* is connected to the *CREB3L2*, *RAB11A*, and *RAB5B*, while in CWM it is connected to no hsa04962 gene. Remarkably, the hsa04962 genes that also have significant independence with respect to *AVP* are*: DYNC1H1* in NOR, *CREB3* and *RAB1A* in PTA, *ARHGDIB* in PTB and *ARHGDIA*, *DCTN1,* and *DYNC1H1* in CWM.

## 4. Discussion

This study continues the analyses from a previous article [40] on four samples collected from the chest wall metastasis (CWM), two primary tumor regions (PTA and PTB), and the surrounding normal tissue (NOR) in the right kidney of a man with metastatic ccRCC. In [40], we analyzed the ccRCC impact on cyclins, cyclin kinases, and the functional pathways of apoptosis, chemokine and VEGF signaling, oxidative phosphorylation, basal transcription factors, and RNA polymerase, while here we focused on the five KEGG-constructed functional pathways of the excretory system.

An important finding of the previous paper [40], reconfirmed in the present study, was that even equally Fuhrman-graded (3) and closely located cancer nodules from the same kidney exhibit substantial transcriptomic differences and are under the command of distinct gene master regulators. Considerably transcriptomic differences between equally graded cancer nodules were also reported by us in two prostate cancer studies [34,42]. Importantly, tumor heterogeneity, present even in patient-derived RCC organoids [64], is not limited to the genes’ mutations [65] or expression levels [66], but encompasses also the control of the transcripts’ abundances and the way the genes are interconnected in functional pathways. Tumor heterogeneity and the unrepeatable set of cancer-favoring factors characterizing each human make the “transcriptomic signature” unreliable (e.g., [67,68]), and even of the integrated proteogenomic characterization [69] derived from comparing genomic data from large populations of cancer and healthy people is made unreliable. Instead, it imposes the normal tissue surrounding the cancer nodule(s) in the tumor as the most trustable reference.

The novel findings on the alterations of the mechanisms controlling the transcript abundances and the remodeling of the functional pathways were possible by adopting the Genomic Fabric Paradigm (GFP) and the use of its mathematically advanced algorithms and software (detailed in [54]). The GFP improves the traditional gene expression analysis by replacing the arbitrarily introduced (1.5×) cut-off for the absolute fold-change of a gene to be considered as significantly regulated with a cut-off computed for each gene in the compared conditions that considers both biological variability and technical noise. While incorporating an improved version of the traditional analysis, the GFP goes further by also analyzing the gene expression control and inter-coordination. These additional characteristics provide as much supplementary information as going from knowing the height of a mountain to featuring it on a 3D scale model. The independence of the three types of expression characteristics was illustrated here for the genes involved in the KEGG-constructed pathway of the “Endocrine and other factor-regulated calcium absorption”. The independence of the three types of expression characteristics can be proven for any other gene subset (principle discussed in [53]). For instance, in previously published cancer genomics papers, we proved the independence of genes involved in: chemokine signaling [40], apoptosis [55], evading apoptosis [42], and mTOR signaling [34].

The gene networks constructed with the COR analysis do not have the same faults of universality, unicity, and rigidity as those built with the KEGG and other software. The COR-determined networks are not universal but instead depend on the race, age, and other personal characteristics of the patient that modulate his/her gene expression, as we proved in [34] for two prostate cancer patients and two standard human prostate cancer cell lines. They are not unique, with cancer nodule(s) and normal tissue exhibiting different gene wiring, and certainly not rigid, but remodeling during aging, the progression of the disease, and in response to treatment and other external stimuli. Thus, the Postulate of Transcriptomic Stoichiometry (PTS, [44]) also extends Dalton’s Law of Multiple Proportions from chemistry [45] to gene networks.

This analysis of the expression correlation among the genes of the “Endocrine and other factor-regulated calcium reabsorption” revealed an interesting partnership with the estrogen receptor 1 (*ESR1*), which is a tumor driver and drug-targeting factor in cancer [70], and regulator of age-related mitochondrial dysfunction and inflammation [71]. Thus, while its expression synergisms with the *ATP2B3* (ATPase, Ca^++^ transporting, plasma membrane 3) and *KLK2* (kallikrein-related peptidase 2) in NOR are not modified by ccRCC, the antagonistic expression with the *AP2A1* is switched to a synergistic one in all three cancer nodules. Because the *AP2A1* is important in vesicle formation and intracellular membrane trafficking [72], our result indicates that cancer switched the type of estrogen influence on the intracellular transport phenomena. Interestingly, the altered expression of *ESR1* was associated with anti-cancer drug resistance, and non-coding events were identified in the regulatory hotspot of *AP2A1* in various metastatic cancers [73].

We found that the excretory genes rank much lower than the GMRs identified for each region in [40]. Thus, the *GNAQ* (guanine nucleotide-binding protein (G protein), q polypeptide), the top gene involved in the “Endocrine and other factor-regulated calcium reabsorption” in both NOR (GCH^(NOR)^ = 9.46) and CWM (GCH^(CWM)^ = 17.43), is far below the GMRs of these regions: the *DAPK3* (death-associated protein kinase 3, GCH^(NOR)^ = 30.31) and the *ALG13* (UDP-N-acetylglucosaminyltransferase subunit, GCH^(CWM)^ = 82.95). The *IGF1* (insulin-like growth factor 1 (somatomedin C), GCH^(PTA)^ = 13.11) from the pathway of the “Aldosterone-regulated sodium reabsorption” is far below the *TASOR* (transcription activation suppressor, GCH^(PTA)^ = 63.97) in PTA. The *ATP1A2* (ATPase, Na^+^/K^+^ transporting, alpha 2 polypeptide, GCH^(PTB)^ = 9.95) from the pathway of the “Proximal tubule bicarbonate reclamation” is below the *FAM27C* (family with sequence similarity 27, member C, GCH^(PTB)^ = 57.19) in PTB. The much lower GCH scores mean that although the excretory pathways were strongly regulated, no excretory gene is an efficient target for gene therapy against any of the three cancer nodules.

The substantial decrease in the homeostatic control of *AP2A1* expression in PTA but not in PTB and CWM indicates that, although not significantly regulated (*x*^(*PTA*→*NOR*)^ = 1.22 < 1.57 = CUT^(*PTA*→*NOR*)^, *x*^(*PTB*→*NOR*)^ = 1.04 < 1.61 = CUT^(*PTB*→*NOR*)^, *x*^(*CWM*→*NOR*)^ = 1.13 < 1.55 = CUT^(*CWM*→*NOR*)^), the *AP2A1* lost its importance for the cell homeostasis in PTA while keeping it at NOR level in the other nodules. The most interesting case for the expression control analysis was that of *ADCY9*, a biomarker for glioma [74], lung [75], and hepatocellular carcinoma [76], as well as colorectal [77], bladder [78], and pancreatic cancers [79]. The *ADCY9* exhibited the largest increase of the homeostatic control in PTB and the largest decrease in CWM among all profiled excretory genes, while its control in PTA remained the same as in the normal tissue. These results indicate that while cancer had no effect on the homeostatic control of the *ADCY9* transcript abundance in one region (PTA) of the tumor, it made the right *ADCY9* expression critical in another region (PTB), and totally irrelevant in the metastasis nodule (CWM). Since the expression of *ADCY9* is normally four times more controlled than the median kidney gene, we predict that the expression manipulation of *ADCY9* will wholly jeopardize the PTB cells, and have a similar (high) negative effect on both normal and PTA cells, while stimulating the proliferation of CWM cells. Thus, at least for this patient, targeting *ADCY9* could be both beneficial and harmful.

The increased overall correlation of the *ADCY6* and *AP2A1* with all other genes from the “Endocrine and other factor-regulated calcium reabsorption” pathway (Figure 3e) in PTA, but the decrease in the other two cancer nodules, indicates substantially different gene networks. Thus, according to Figure 6, in NOR, the *AP2A1* has four statistically significant synergistically (*ADCY6*, *DNM2*, ***FXYD2***, *PLCB1*) and four antagonistically (***ATP1A2***, ***ATP2B3***, ***ESR1***, ***KLK2***) expressed partners, while in PTA it has eleven synergistic (*ADCY6*, ***ATP1A2***, *ATP1A4*, ***ATP2B3***, *CLTCL1*, ***ESR1***, *GNAQ*, *GNAS*, ***KLK2***, *PRKCB*, *VDR*) and five antagonistic (*ATP2M1*, *ATP1B3*, *CLTA*, ***FXYD2***) partners (bolded symbols indicate the genes that switched the expression correlation type in PTA with respect to NOR). The partnerships of *AP2A1* were partially similar in PTB: it had synergism with *ATP2B3*, *CLTB*, *CLTCL1*, *ESR1*, and *GNAQ*, and antagonism with *AP2S1*, *ATP1B1*, *ATP1B3*, *PLCB2*, *PRKCA*, and S*LC8A1* (underlined symbols indicate common partners in PTA and PTB).

Because *ADCY6* is a promising target in cancer therapy [80], it is important to know how it relates to other excretory genes. Thus, in normal tissue (NOR) it is synergistically expressed with *AP2A1* and *ATP1B1*, and antagonistically expressed with *ATP1A2*, *ATP2B3*, *ESR1*, and *KLK2*, as well as independently expressed with *CLTB.* In PTA, the synergism with *AP2A1* is preserved but that with *ATP1B1* is switched to antagonism. PTA adds synergism with *GNAQ*, *GNAS*, and *VDR* and antagonism with *AP2A2* and *ATP1B3.* The overall coordination of *ADCY6* with other genes from the pathway is diminished in PTB (synergism with *ATP1A3* and antagonism with *BDKRB2*, *CLTA*, and PLCB2) and CWM (synergism with *ATP1A4* and *KL*, and antagonism with *ATP1A3* and *FXYD2*). Therefore, for this patient, manipulating the expression of *ADCY6* would have different effects on his cancer nodules.

The down-regulation of *AQP3* (x = −3.889) was reported recently [81] by performing a meta-analysis of public datasets from the publicly accessible databases of ONCOMINE [82] and UALCAN [83]. In the cited study, the authors compared the expressions of all aquaporin encoding genes (*AQP1*/*2*/*3/4*/*5*/*6*/*7/8*/*9*/*10*/*11*) in 533 tumor cases with 72 normal cases, using the uniform fold-change threshold of 1.50× and *p*-value < 0.01, to decide whether a gene was significantly regulated. Since no web resource specifies the exact locations in the kidney from which the samples have been collected, nor whether the tumors contained more cancer nodules, the authors implicitly assumed homogeneous gene expressions all over the tumor. In contrast, our results (*AQP3* down-regulated in PTA and CWM but up-regulated in PTB) indicate that ccRCC tumors do not exhibit uniform but rather heterogeneous gene expressions, and therefore the meta-analyses comparing cancer cases with healthy cases have little biological relevance beyond the statistics exercise. Instead, the best reference for cancer nodules is the normal tissue still present in the tumor.

Figure 7 shows distinct statistically significant coordination partners of *AVP*, the neuropeptide hormone arginine vasopressin, which is a very important regulator of kidney salt and water homeostasis [84]. Our analysis detailed also how *AVP*-dependent water reabsorption regulates the cAMP signaling pathway [85,86] through expression correlation with genes shared by the cAMP signaling pathway with the excretory pathways. Thus, in NOR, AVP is synergistically expressed with *ATP1B2*, *CREB3L1*, *CREB3L4*, and *FXYD2*, has no antagonistic partners, and is independently expressed with *PIK3CB.* In PTA, *AVP* is synergistically expressed with *ATP1B2* and *CREB3L3*, antagonistically expressed with *ATP1A1* and *PRKACB*, and independently expressed with *ATP1A4*, *CREB3*, and *MAPK1.* In PTB, *AVP* is synergistically expressed with *CREB3L2* and independently expressed with *ATP1A3*, while in CWM it is synergistically expressed with *ATP1A2* and antagonistically expressed with *PIK3R1. AVP* is also involved in the KEGG-constructed pathways of hsa04270 “Vascular smooth muscle contraction”, and the very important hsa04020 “Calcium signaling pathway” and hsa04072 “Phospholipase signaling pathway”.

Interestingly, *CREB3L4*, known for its role in proliferating prostate cancer cells [87], was significantly down-regulated in all kidney cancer nodules (x^(PTA)^ = −1.40, x^(PTB)^ = −1.74, x^(CWM)^ = −1.95). Out of 18 regulated excretory genes in PTA and twenty-one in PTB, only five were similarly regulated (*ATP1B2*, *CREB3L4*, *DCTN2*, *PIK3R2*, *SLC4A4*) and one (*AQ3*) was oppositely regulated.

Altogether, the differences in gene expression regulation and remodeling of the excretory pathways among the three cancer nodules indicate that the bio-assays used to identify the ccRCC presence by the regulation of certain gene biomarkers might not be always valid. For instance, the *CA9* (carbonic anhydrase IX) that should be expressed only in ccRCC [88,89] was found by us to be expressed not only in the cancer nodules (*AVE*^(*PTA*)^ = 2.11, *AVE*^(*PTB*)^ = 0.27, *AVE*^(*CWM*)^ = 2.65) but also in the NOR (*AVE*^(*NOR*)^ = 2.76). Moreover, the *CA9* exhibited a significant down-regulation in PTB (x^(PTB→NOR)^ = −10.37).

Nonetheless, adoption of the GFP increases by 3–4 orders of magnitude the computational effort, thus opening the field of genomics to artificial intelligence applications [90] that have the power to combine practically unlimited amounts of histo-pathological, imaging and “omic” information [91].

## 5. Conclusions

Although based on a single metastatic ccRCC case, our study shows that adding the analyses of transcriptomic control and expression inter-coordination of the genes provides a significantly deeper understanding of cancer-related transcriptomic alterations. Going from traditional unidimensional gene expression analysis to the GFP is like going from determining only the numbers of electronic items of each type needed to fix a complex robot, to knowing also how to wire these items and what voltages to apply to each of them. Thus, the GFP not only incorporates the traditional gene expression analysis but complements it with a detailed examination of the expression control and gene inter-coordination, altogether providing a complete characterization of the transcriptome.

The unrepeatable combination of cancer-favoring factors among humans questions the validity of the cancer biomarkers derived from meta-analyses of large genomic datasets. The intra-tumor transcriptomic heterogeneity makes the transcriptomic signatures even less reliable. Therefore, we believe that the personalization of cancer gene therapy should go beyond the patient to the primary cancer clones present in his/her tumor. The progress of gene-editing technology will soon allow the industry to produce silencing constructs for all genes at reasonable prices so that personalized cancer gene therapy will become affordable.

## Figures and Tables

**Table 1:** Numbers of mutated genes in 14 primary sites (data from [20])**.** Note that the number of mutated genes in the listed individual sites represents from 86.94% (prostate cancer) to 95.07% (bone marrow cancer) of the 22,588 mutated genes reported in all 88,991 cancer cases located in all 68 primary sites.

Primary Site	# of Cases	# of Genes	Protein Coding	# of Mutations	Primary Site	# of Cases	# of Genes	Protein Coding	# of Mutations
Bladder	1725	20,183	19,692	114,662	Lung	12,262	21,318	19,790	443,974
Bone marrow	11,027	21,474	19,705	163,756	Ovary	3381	20,266	19,673	64,142
Brain	1452	20,343	19,729	93,128	Pancreas	2776	19,874	19,502	36,676
Breast	9121	20,454	19,727	113,777	Prostate	2387	19,638	19,402	27,468
Colorectal	8140	21,060	19,794	337,634	Skin	2893	20,739	19,770	353,213
Head & neck	2792	20,535	19,712	116,274	Stomach	1631	20,336	19,739	182,493
Kidney	3501	20,129	19,631	65,471	Uterus	2803	21,471	19,781	769,622

**Table 2:** Number of quantified out of number of included genes in the five KEGG-constructed functional pathways of the excretory system, their percentages, and overall weighted regulations in each cancer nodule. ALDO = Aldosterone-regulated sodium reabsorption, COLL = Collecting duct acid secretion, ENDO = Endocrine and other factor-regulated calcium reabsorption, PROX = Proximal tubule bicarbonate reclamation, and VASO = Vasopressin-regulated water reabsorption.

		PTA			PTB			CWM		
Path	Genes	%Up	%Down	WPR	%Up	%Down	WPR	%Up	%Down	WPR
ALDO	26/37	11.54	11.54	1.12	30.77	0.00	8.19	11.54	11.54	2.32
COLL	16/27	6.25	12.50	5.20	12.50	0.00	16.36	12.50	0.00	9.69
ENDO	37/53	5.41	2.70	0.88	18.92	5.41	7.68	8.11	5.41	2.15
PROX	18/23	16.67	0.00	0.96	38.89	0.00	9.04	11.11	0.00	2.12
VASO	36/44	8.33	11.11	0.69	11.11	5.56	0.93	2.78	8.33	0.77

## Data Availability

Transcriptomic data are publicly available at https://www.ncbi.nlm.nih.gov/geo/query/acc.cgi?acc=GSE72304 (accessed on 26 October 2023).

## Supplementary Materials

The following supporting information can be downloaded at: https://www.mdpi.com/article/10.3390/cimb45120594/s1, Figure S1: Regulated genes in the KEGG-constructed functional pathway hsa04960 Aldosterone-regulated sodium reabsorption, Figure S2: Regulated genes in the KEGG-constructed functional pathway hsa04966 Collecting duct acid secretion, Figure S3: Regulated genes in the KEGG-constructed functional pathway hsa04961 Endocrine and other factor-regulated calcium reabsorption, Figure S4: Regulated genes in the KEGG-constructed functional pathway hsa04964 Proximal tubule bicarbonate reclamation.

## Appendix A

1. **Normalized gene expression levels in the biological replica “k” in condition “c”:**
  (A1) $$\forall c=PTA,PTB,CWM,NOR\;\&\;k=1÷4\;\alpha_{i}^{\left(c;k\right)}\equiv \frac{a_{i}^{\left(c;k\right)}}{{\langle a_{j}^{\left(c;k\right)}\rangle }_{j=1÷N}}\Rightarrow {\langle \alpha_{j}^{\left(c;k\right)}\rangle }_{j=1÷N}=1$$
  *a_i_*^(*c*;*k*)^ is the sum of the net fluorescences of all microarray spots probing gene “*i*” in the biological replica “*k*” of condition “*c*”.

2. **Relative expression variation:**
  (A2) $$\begin{array}{l} REV_{i}^{\left(c\right)}(\alpha )\equiv \frac{\sigma_{i}^{\left(c\right)}}{2AVE_{i}^{\left(c\right)}}\left(\sqrt{\frac{r_{i}}{\chi^{2}(\beta ;r)}}+\sqrt{\frac{r_{i}}{\chi^{2}(1-\beta ;r)}}\right)\times 100\%\;,\;where:\\ \sigma_{i}^{\left(c\right)}=sdev{\left(\alpha_{i}^{\left(c;k\right)}\right)}_{k=1÷4},\;\beta \;(\mathrm{usually}\;\beta =0.05)\;\mathrm{is}\;\mathrm{the}\;\mathrm{probability},\\ r_{i}\;\mathrm{is}\;\mathrm{the}\;\mathrm{number}\;\mathrm{of}\;\mathrm{degrees}\;\mathrm{of}\;\mathrm{freedom},\;r_{i}=n\nu_{i}-1 \end{array}$$
  and *χ^2^* is the chi-square score with *β* probability for *r* degrees of freedom.

3. **Transcriptome configuration function:**
  $$F(1,2,\ldots ,N)=A_{1}\underset{\mathrm{independent}}{\underset{︸}{\prod_{i=1}^{N}f_{1}(i)}}+A_{2}\underset{\mathrm{pair}-\mathrm{wise}}{\underset{︸}{\prod_{i>j=1}^{N}f_{2}(i,j)}}+A_{3}\underset{3-\mathrm{genes}\;\mathrm{clusters}}{\underset{︸}{\prod_{i>j>k=1}^{N}f_{3}(i,j,k)}}+\ldots +A_{N}\underset{\mathrm{all}\;\mathrm{genes}\;\mathrm{cluster}}{\underset{︸}{f_{N}(1,2,\ldots ,N)}}$$
  where *A*_1_, …, *A_N_* are the probabilities of each configuration of gene clustering, and *f*_2_, *f*_3_, …, *f_N_* are distribution functions symmetrical to the permutation(s) of the genes. The above expansion satisfies the following conditions:
  $$\begin{array}{l} \mathrm{probability}\;\mathrm{condition}:\;\sum_{p=1}^{N}A_{p}=1\;\\ \mathrm{norm}\;\mathrm{conditions}:\;\\ \int F(1,2,\ldots ,N)dV=1\;,\;\forall p=1÷N\;\to \;\int_{V}\left(\prod_{i_{1}>i_{2}>\ldots >i_{p}=1}^{N}f_{p}(i_{1},i_{2},\ldots ,i_{p})\right)dV=1 \end{array}$$

A 3-gene cluster can be approximated with the superposition of three paired genes:

$$\begin{array}{l} \prod_{i>j>k}^{N}f_{3}(i,j,k)≃\prod_{i>j>k}^{N}f_{2}(i,j)f_{2}(j,k)f_{2}(k,i) \end{array}$$

A 4-gene cluster can be approximated with four 3-gene clusters:

$$f_{4}(i,j,k,l)=f_{3}(i,j,k)f_{3}(j,k,l)f_{3}(k,l,i)f_{3}(l,i,j)$$

that can be further approximated with six squared distributions of paired genes:

$$\begin{array}{l} f_{4}(i,j,k,l)=\underset{f_{3}(i,j,k)}{\underset{︸}{\left(f_{2}(i,j)f_{2}(j,k)f_{2}(k,i)\right)}}\times \underset{f_{3}(j,k,l)}{\underset{︸}{\left(f_{2}(j,k)f_{2}(k,l)f_{2}(l,j)\right)}}\times \underset{f_{3}(k,l,i)}{\underset{︸}{\left(f_{2}(k,l)f_{2}(l,i)f_{2}(i,k)\right)}}\\ \times \underset{f_{3}(l,i,j)}{\underset{︸}{\left(f_{2}(i,j)f_{2}(j,l)f_{2}(l,i)\right)}}=f_{2}^{2}(i,j)f_{2}^{2}(j,k)f_{2}^{2}(k,i)f_{2}^{2}(k,l)f_{2}^{2}(l,j)f_{2}^{2}(l,i) \end{array}$$

and so on, giving the recurrence relation:

$$\begin{array}{l} \forall p\geq 3\;\&\;I_{p}=a\;\mathrm{set}\;\mathrm{of}\;p\;\mathrm{genes}\\ f_{p}(I_{p})≃\prod_{\begin{array}{c} \mathrm{all}\;\mathrm{combinations}\;\mathrm{of}\;p\;\mathrm{genes}\\ \mathrm{in}\;\mathrm{sets}\;\mathrm{of}\;p-1\;\mathrm{genes} \end{array}}f_{p-1}(I_{p-1})≃\prod_{i>j\in I_{p}}f_{2}^{p-2}(i,j) \end{array}$$

From the normalization condition, it results that:

$$\forall p\geq 3\;\wedge \;\forall q>r=1÷N\;,\;\prod_{i>j\in I_{p}}f_{2}^{p-2}(i,j)<<\prod_{i>j\in I_{p}}f_{2}(i,j)\;$$

Therefore, one can neglect the contributions of clusters with more than 2 genes:

(A3) $$F(1,2,\ldots ,N)\approx A_{1}\underset{\mathrm{independent}}{\underset{︸}{\prod_{i=1}^{N}f_{1}(i)}}+A_{2}\underset{\mathrm{pair}-\mathrm{wise}}{\underset{︸}{\prod_{i>j=1}^{N}f_{2}(i,j)}}$$

4. **Pair-wise correlation of gene expression levels:**

(A4)
$$COR_{i,j}^{\left(c\right)}\equiv \frac{\sum_{k=1}^{4}\left(\log_{2}\alpha_{i}^{\left(c;k\right)}-\log_{2}AVE_{i}^{\left(c\right)}\right)\left(\log_{2}\alpha_{j}^{\left(c;k\right)}-\log_{2}AVE_{j}^{\left(c\right)}\right)}{\sqrt{\sum_{k=1}^{4}{\left(\log_{2}\alpha_{i}^{\left(c;k\right)}-\log_{2}AVE_{i}^{\left(c\right)}\right)}^{2}}\sqrt{\sum_{k=1}^{4}{\left(\log_{2}\alpha_{j}^{\left(c;k\right)}-\log_{2}AVE_{j}^{\left(c\right)}\right)}^{2}}}$$

5. **Gene Commanding Height:**

(A5)
$${GCH}_{i}^{\left(c\right)}\equiv \frac{{\langle REV\rangle }^{\left(c\right)}}{{REV}_{i}^{\left(c\right)}}\times exp\left(4\bar{{{COR}_{i,j}^{\left(c\right)}}^{2}}\right)$$

6. **Statistically significant regulation of the expression level:**

(A6)
$$\begin{array}{l} Abs\left(x_{i}^{\left(cancer\to NOR\right)}\right)\geq CUT_{i}^{\left(cancer\to NOR\right)}=1+\sqrt{2\left({\left(\frac{REV_{i}^{\left(cancer\right)}}{100}\right)}^{2}+{\left(\frac{REV_{i}^{\left(NOR\right)}}{100}\right)}^{2}\right)}\;\&\;p_{i}^{\left(cancer\to NOR\right)}<0.05\\ \mathrm{where}:\;x_{i}^{\left(cancer\to NOR\right)}=\left\{\begin{array}{ll} \frac{\;\;\;AVE_{i}^{\left(cancer\right)}}{AVE_{i}^{\left(NOR\right)}}\;\;\;\;\;\;\;\;if & AVE_{i}^{\left(cancer\right)}\geq AVE_{i}^{\left(NOR\right)}\\ -\frac{AVE_{i}^{\left(NOR\right)}}{AVE_{i}^{\left(cancer\right)}}\;\;\;\;\;\;if & AVE_{i}^{\left(cancer\right)}<AVE_{i}^{\left(NOR\right)} \end{array}\right.\;,\;cancer=PTA,PTB,CWM \end{array}$$

7. **Weighted Individual (Gene) Regulation:**

(A7)
$$WIR_{i}^{\left(cancer\to NOR\right)}\equiv AVE_{i}^{\left(NOR\right)}\frac{x_{i}^{\left(cancer\to NOR\right)}}{Abs\left(x_{i}^{\left(cancer\to NOR\right)}\right)}\underset{\mathrm{absolute}\;\mathrm{fold}-\mathrm{change}}{\underset{︸}{\left(Abs\left(x_{i}^{\left(cancer\to NOR\right)}\right)-1\right)}}\underset{\mathrm{confidence}}{\underset{︸}{\left(1-p_{i}^{\left(cancer\to NOR\right)}\right)}}$$

8. **Weighted Pathway Regulation:**

(A8)
$${WPR}_{\Gamma }^{\left(cancer\to NOR\right)}={\left.\bar{Abs({WIR}_{i}^{\left(cancer\to NOR\right)})}\right\rfloor }_{i\epsilon \Gamma }$$

9. **Relative Expression Control:**

(A9)
$$REC_{i}^{\left(condition\right)}\equiv \left(\frac{\langle REV^{\left(condition\right)}\rangle }{REV_{i}^{\left(condition\right)}}-1\right)\times 100\%\;,\;\mathrm{where}\;\langle REV^{\left(condition\right)}\rangle \;\mathrm{is}\;\mathrm{the}\;\mathrm{median}\;REV$$

10. **Regulation of the Expression Control:**

(A10)
$$\Delta REC_{i}^{\left(cancer\to NOR\right)}\equiv \frac{const}{REV_{i}^{\left(cancer\right)}}-\frac{const}{REV_{i}^{\left(NOR\right)}}\;,\;const=\mathrm{calibration}\;\mathrm{constant}$$

In this study *const* = 100.

11. **Regulation of the Expression Coordination:**

(A11)
$$\Delta COR_{i,\Gamma }^{\left(cancer\to NOR\right)}\equiv \frac{\sum_{j\in \Gamma }\left(COR_{i,j}^{\left(cancer\right)}-COR_{i,j}^{\left(NOR\right)}\right)}{Abs\left(\sum_{j\in \Gamma }\left(COR_{i,j}^{\left(cancer\right)}-COR_{i,j}^{\left(NOR\right)}\right)\right)}\sum_{j\in \Gamma }{\left(COR_{i,j}^{\left(cancer\right)}-COR_{i,j}^{\left(NOR\right)}\right)}^{2}$$

12. **Transcriptomic Distance:**

(A12)
$$TDI_{i,\Gamma }^{\left(cancer\to NOR\right)}\equiv \sqrt{{\underset{\begin{array}{c} \mathrm{regulation}\;\mathrm{of}\\ \mathrm{expression}\;\mathrm{level} \end{array}}{\underset{︸}{\left(WIR_{i}^{\left(cancer\to NOR\right)}\right)}}}^{2}+{\underset{\begin{array}{c} \mathrm{regulation}\;\mathrm{of}\\ \mathrm{transription}\;\mathrm{control} \end{array}}{\underset{︸}{\left(\Delta REC_{i}^{\left(cancer\to NOR\right)}\right)}}}^{2}+\sum_{j\in \Gamma }{\underset{\begin{array}{c} \mathrm{regulation}\;\mathrm{of}\\ \mathrm{expression}\;\mathrm{coordination} \end{array}}{\underset{︸}{\left(COR_{i,j}^{\left(cancer\right)}-COR_{i,j}^{\left(NOR\right)}\right)}}}^{2}}$$
