# Supplementary material for: Genomic Fabrics of the Excretory System’s Functional Pathways Remodeled in Clear Cell Renal Cell Carcinoma
Source: Curr Issues Mol Biol. 2023 Nov 24;45(12):9471–99. doi: 10.3390/cimb45120594 (PMC10742519; doi:10.3390/cimb45120594)
Supplement: Supplementary file 1 [file cimb-45-00594-s001.zip › cimb-2699744-SI.pdf]

current issues in molecular biology

*Article*

# Genomic Fabrics of the Excretory System's Functional Pathways Are Remodeled in Clear Cell Renal Cell Carcinoma

**Dumitru Andrei Iacobas, Ehiguese Alade Obiomon and Sanda Iacobas**

**Supplementary Material**

**Supplementary Figure S1:** Regulated genes in the KEGG-constructed functional pathway hsa04960 Aldosterone-regulated sodium reabsorption. **(a)** PTA nodule. **(b)** PTB nodule. **(c)** CWM nodule. **Regulated genes:** *ATP1A1/A2/A3/A4* (ATPase, Na+/K+ transporting, alpha 1/2/3/4 polypeptide), *ATP1B2/B3* (ATPase, Na+/K+ transporting, beta 2/3 polypeptide), *FXYD3* (FXYD domain containing ion transport regulator 3), *HSD11B2* (hydroxysteroid (11-beta) dehydrogenase 2), IRS1 (insulin receptor substrate 1), *KRAS* (Kirsten rat sarcoma viral oncogene homolog), *PIK3CD* (phosphatidylino- 11 sitol-4,5-bisphosphate 3-kinase, catalytic subunit delta), *PIK3R1/2* (phosphoinositide-3-kinase, regulatory subunit 1 (al- 12 pha)/ 2 (beta), SFN (stratifin).

**(a) PTA**

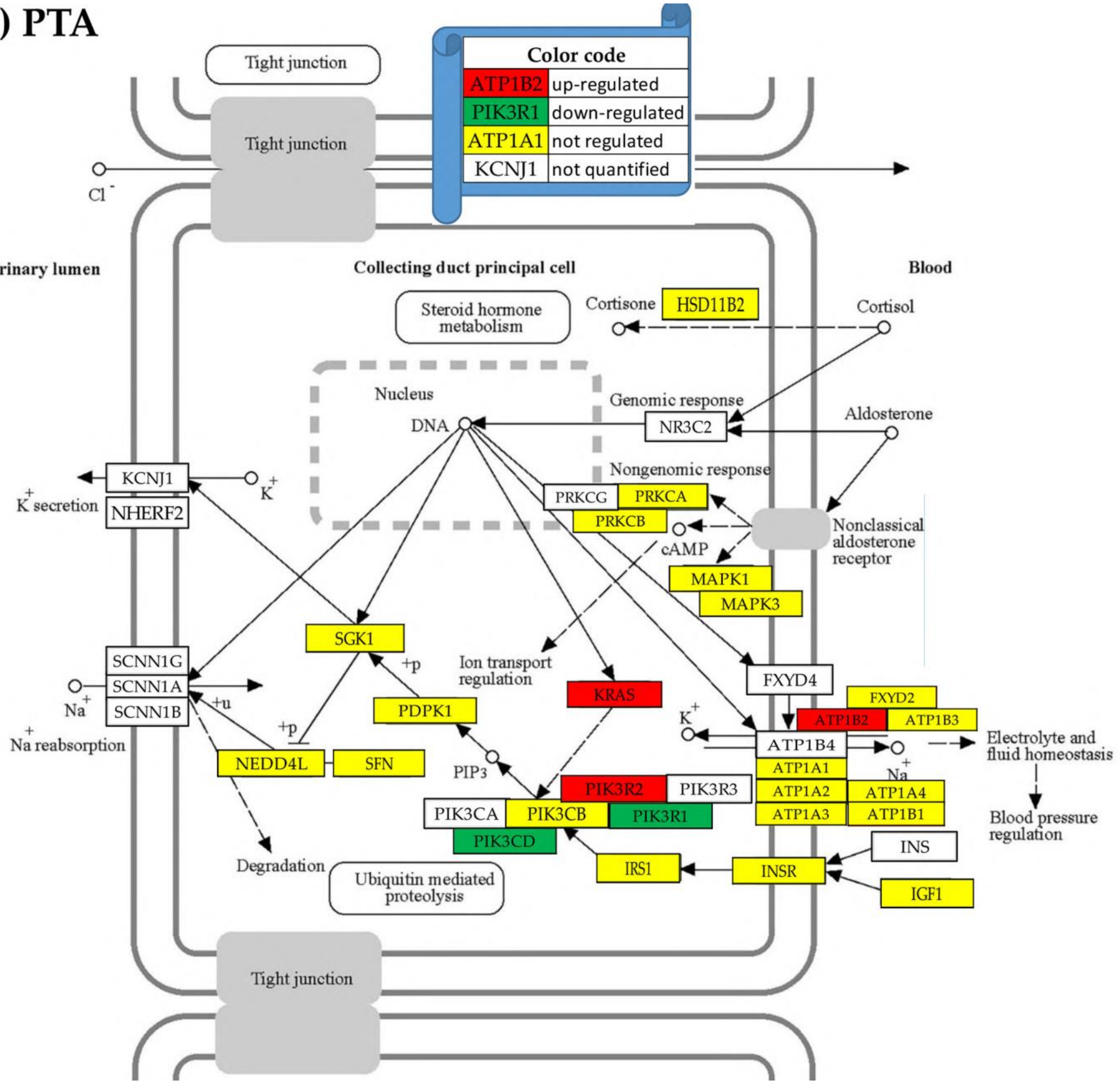

## (b) PTB

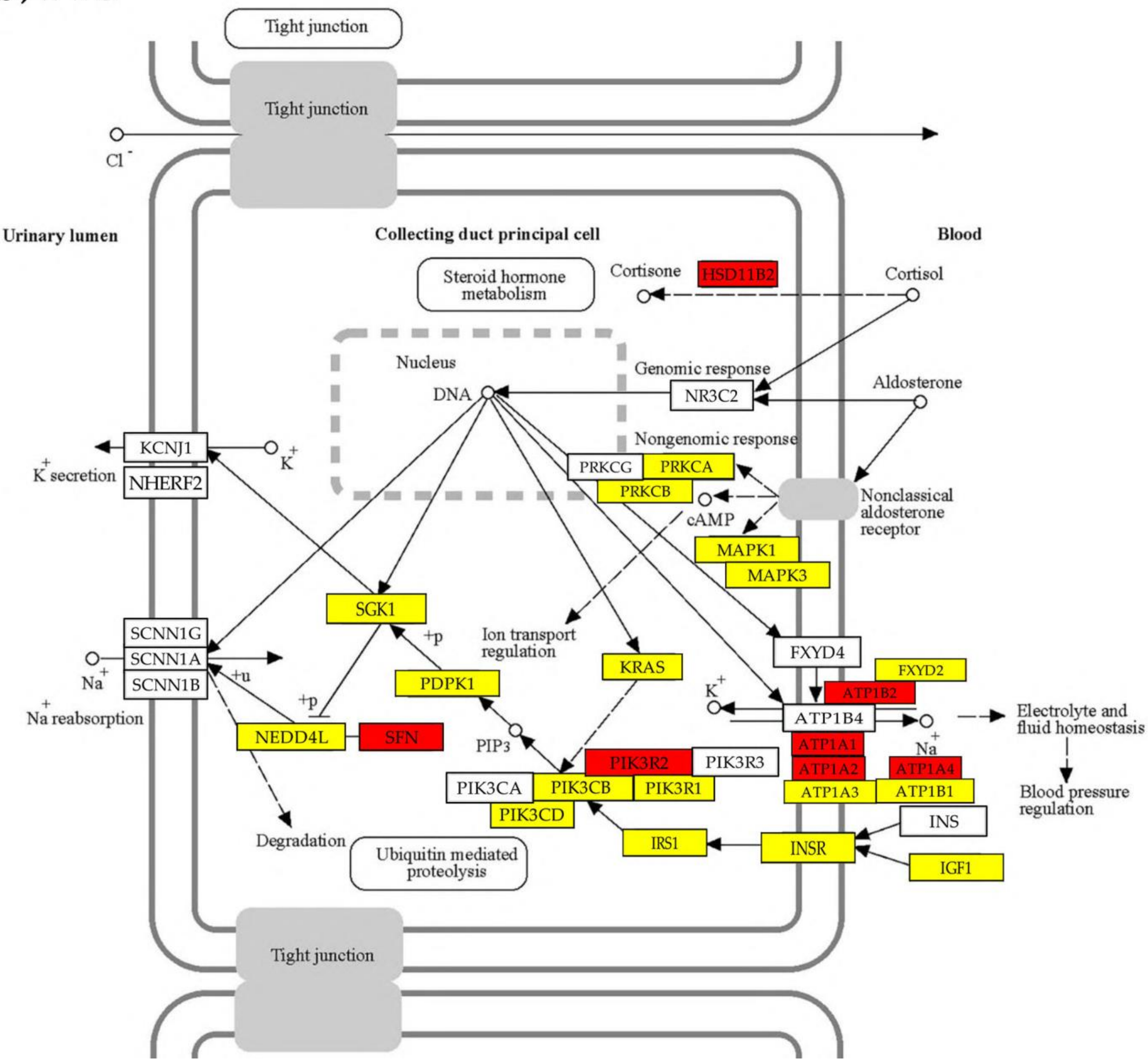

Tight junction
Tight junction
Cl-
Urinary lumen
Collecting duct principal cell
Blood
Steroid hormone metabolism
Cortisone
HSD11B2
Cortisol
Nucleus
DNA
Genomic response
NR3C2
Aldosterone
Nongenomic response
KCNJ1
K+
K+ secretion
NHERF2
PRKCG
PRKCA
PRKCB
cAMP
Nonclassical aldosterone receptor
MAPK1
MAPK3
SGK1
+p
Ion transport regulation
SCNN1G
SCNN1A
SCNN1B
Na+
Na+ reabsorption
+u
+p
PDPK1
KRAS
FXYD4
FXYD2
ATP1B2
K+
ATP1B4
Electrolyte and fluid homeostasis
NEDD4L
SFN
PIP3
PIK3R2
PIK3R3
ATP1A1
Na+
ATP1A2
ATP1A4
PIK3CA
PIK3CB
PIK3R1
ATP1A3
ATP1B1
Blood pressure regulation
PIK3CD
INS
Degradation
Ubiquitin mediated proteolysis
IRS1
INSR
IGF1
Tight junction

**(c) CWM**

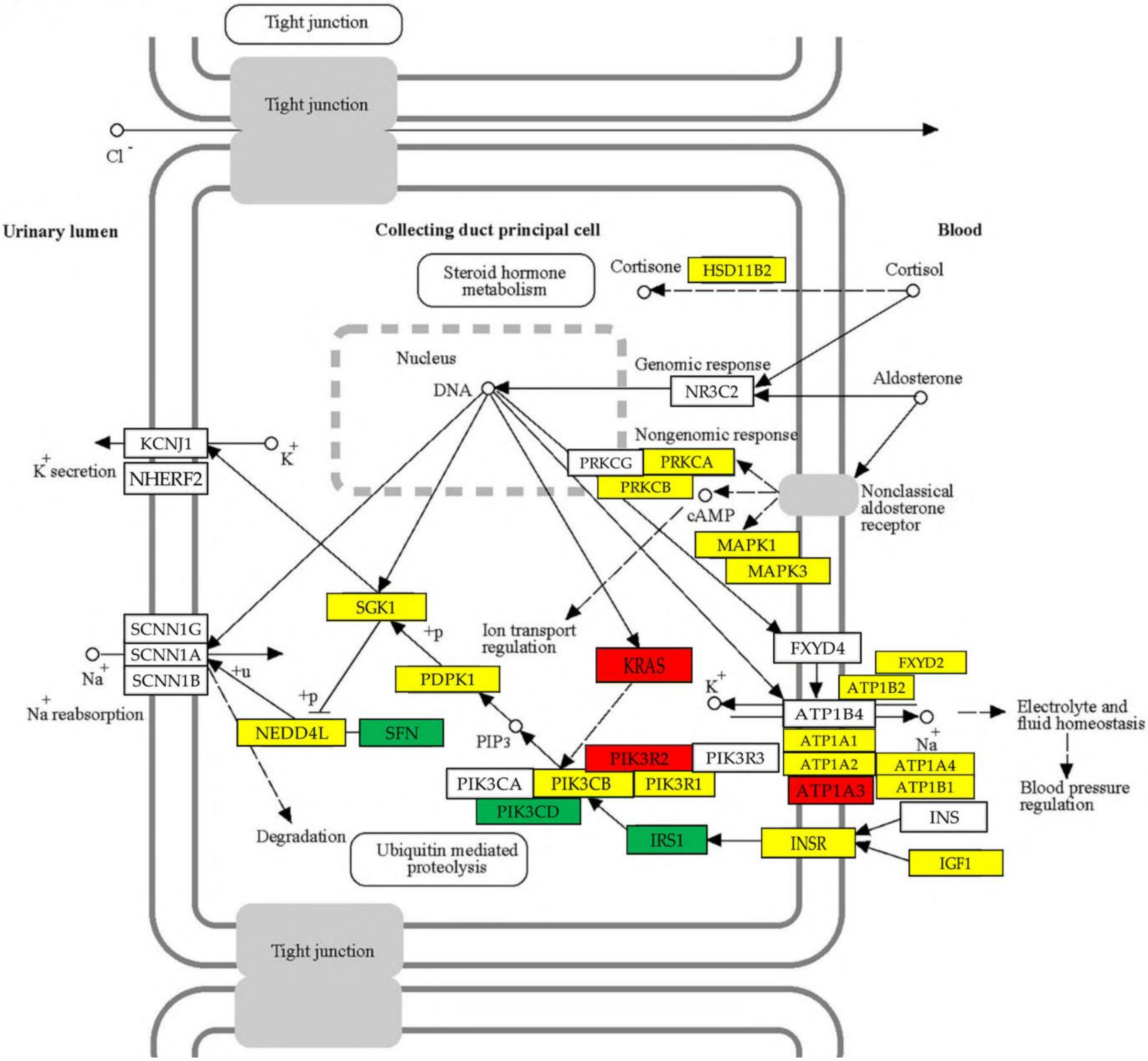

Tight junction
Tight junction
Cl-
Tight junction
Urinary lumen
Collecting duct principal cell
Blood
Steroid hormone metabolism
Cortisone
HSD11B2
Cortisol
Nucleus
DNA
Genomic response
NR3C2
Aldosterone
Nongenomic response
KCNJ1
K+
K+ secretion
NHERF2
PRKCG
PRKCA
PRKCB
cAMP
Nonclassical aldosterone receptor
MAPK1
MAPK3
SGK1
+p
Ion transport regulation
SCNN1G
SCNN1A
SCNN1B
Na+
+u
Na+ reabsorption
PDPK1
KRAS
FXYD4
FXYD2
ATP1B2
K+
ATP1B4
Na+
Electrolyte and fluid homeostasis
+p
NEDD4L
SFN
PIP3
PIK3R2
PIK3R3
ATP1A1
ATP1A2
ATP1A4
PIK3CA
PIK3CB
PIK3R1
ATP1A3
ATP1B1
Blood pressure regulation
PIK3CD
INS
Degradation
Ubiquitin mediated proteolysis
IRS1
INSR
IGF1
Tight junction

**Supplementary Figure S2:** Regulated genes in the KEGG-constructed functional pathway hsa04966 "Collecting duct acid secretion". (a) PTA nodule. (b) PTB nodule. (c) CWM nodule. Regulated genes: ATP6V1A/B2/C1 (ATPase, H+ transporting, lysosomal 70kDa, V1 subunit A/B2/C1), CA2 (carbonic anhydrase II), SLC12A7 (solute carrier family 12 (potassium/chloride transporter), member 7), SLC4A1 (solute carrier family 4 (anion exchanger), member 1 (Diego blood group)).

**(a) PTA**

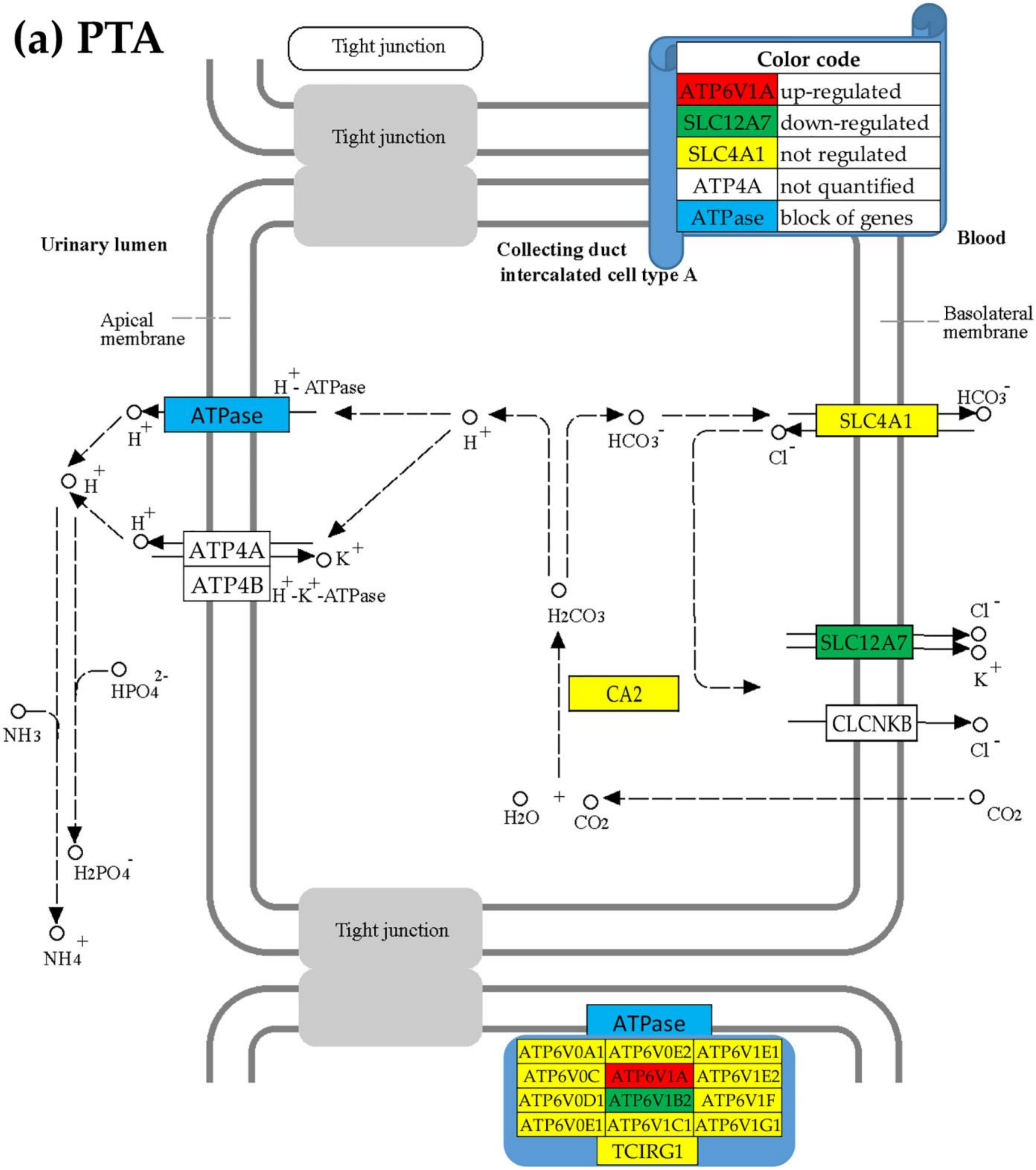

# (b) PTB

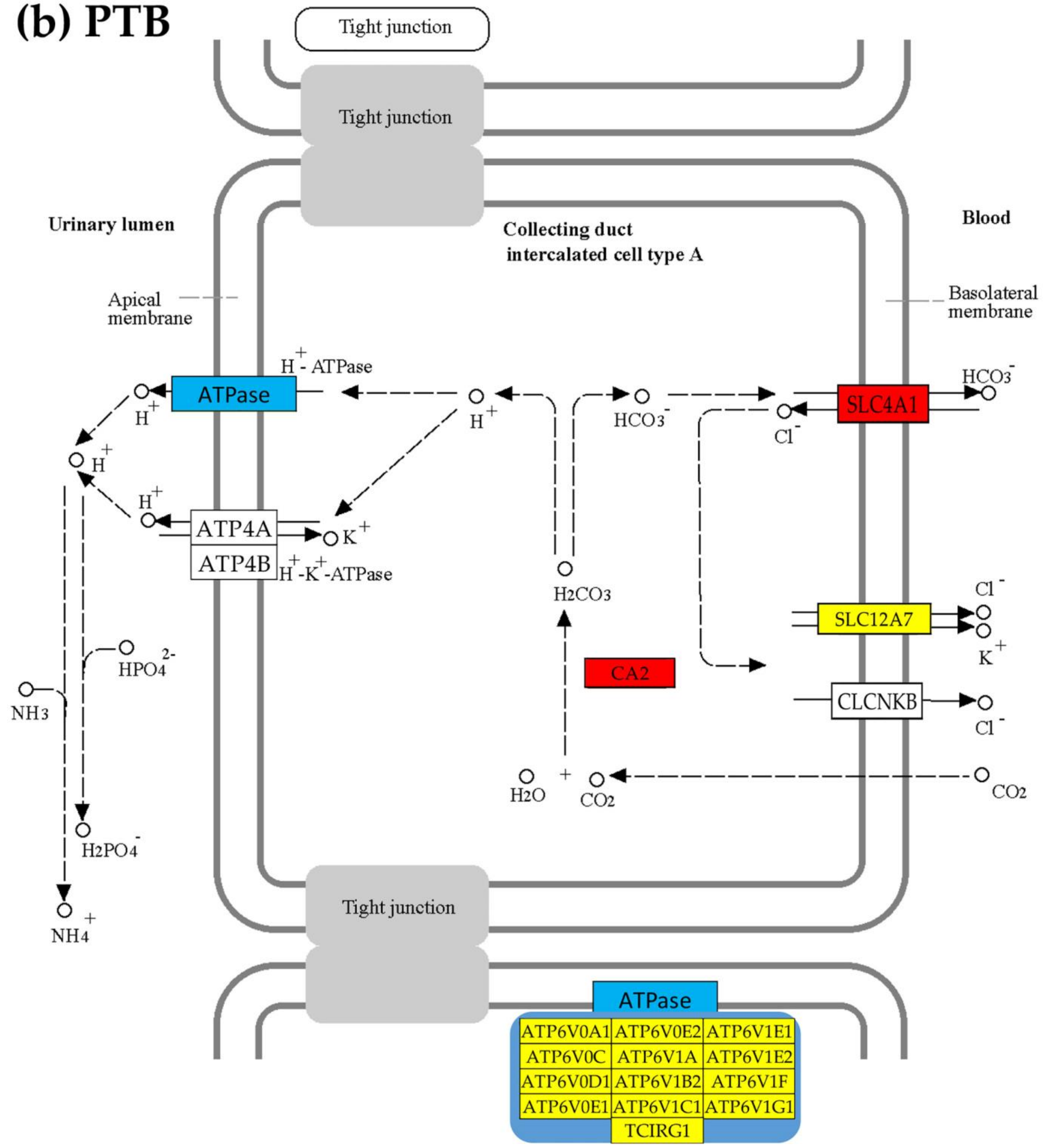

Tight junction
Tight junction
Urinary lumen
Collecting duct
intercalated cell type A
Blood
Apical
membrane
Basolateral
membrane
H+- ATPase
ATPase
H+
H+
H+
HCO3-
SLC4A1
HCO3-
Cl-
H+
ATP4A
ATP4B
K+
H+-K+-ATPase
H2CO3
SLC12A7
Cl-
K+
HPO4 2-
CA2
NH3
CLCNKB
Cl-
H2O
+
CO2
CO2
H2PO4-
NH4+
Tight junction
ATPase
ATP6V0A1
ATP6V0E2
ATP6V1E1
ATP6V0C
ATP6V1A
ATP6V1E2
ATP6V0D1
ATP6V1B2
ATP6V1F
ATP6V0E1
ATP6V1C1
ATP6V1G1
TCIRG1

(c) CWM

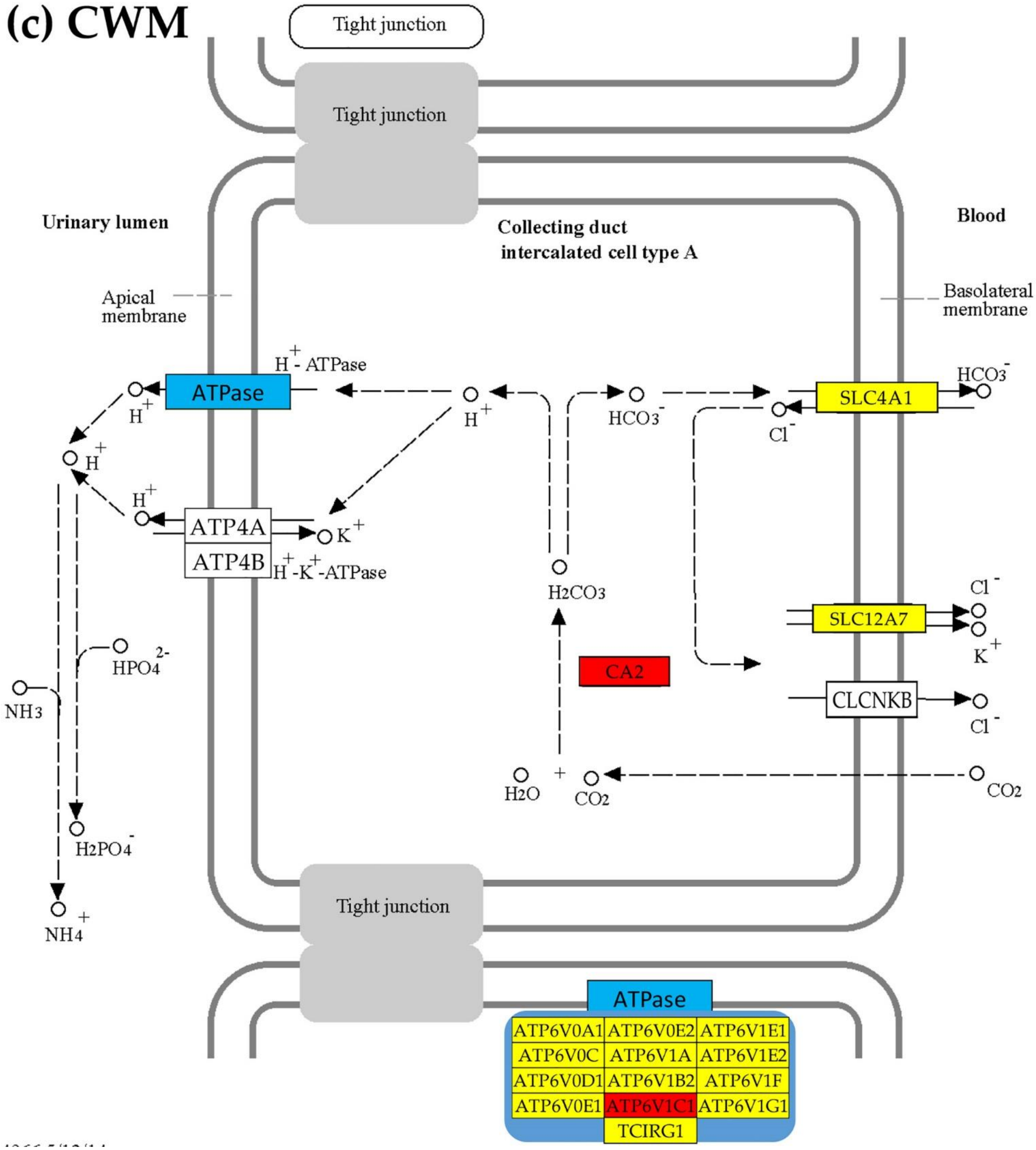
Tight junction
Tight junction
Urinary lumen
Collecting duct
intercalated cell type A
Blood
Apical
membrane
Basolateral
membrane
H+ - ATPase
ATPase
H+
H+
HCO3-
SLC4A1
HCO3-
Cl-
H+
H+
ATP4A
ATP4B
K+
H+-K+-ATPase
H2CO3
Cl-
SLC12A7
K+
CA2
CLCNKB
Cl-
HPO4 2-
NH3
H2O
+
CO2
CO2
H2PO4-
NH4+
Tight junction
ATPase
ATP6V0A1
ATP6V0E2
ATP6V1E1
ATP6V0C
ATP6V1A
ATP6V1E2
ATP6V0D1
ATP6V1B2
ATP6V1F
ATP6V0E1
ATP6V1C1
ATP6V1G1
TCIRG1

**Supplementary Figure S3:** Regulated genes in the KEGG-constructed functional pathway hsa04961 "Endocrine and other factor-regulated calcium reabsorption". **(a)** PTA nodule. **(b)** PTB nodule. **(c)** CWM nodule. Regulated genes: *ADCY6* (adenylate cyclase 6), *ATP1A1/2/3/4* (ATPase, Na+/K+ transporting, alpha 1/2/3/4 polypeptide), *ATP1B2/3* (ATPase, Na+/K+ transporting, beta 2/3 polypeptide), *BDKRB2* (bradykinin receptor B2), *GNAS* (GNAS complex locus), *PLCB1* (phospholipase C, beta 1 (phosphoinositide-specific)), *SLC8A1* (solute carrier family 8 (sodium/calcium exchanger), member 1), *VDR* (vitamin D (1,25- dihydroxyvitamin D3) receptor).

**(a) PTA**

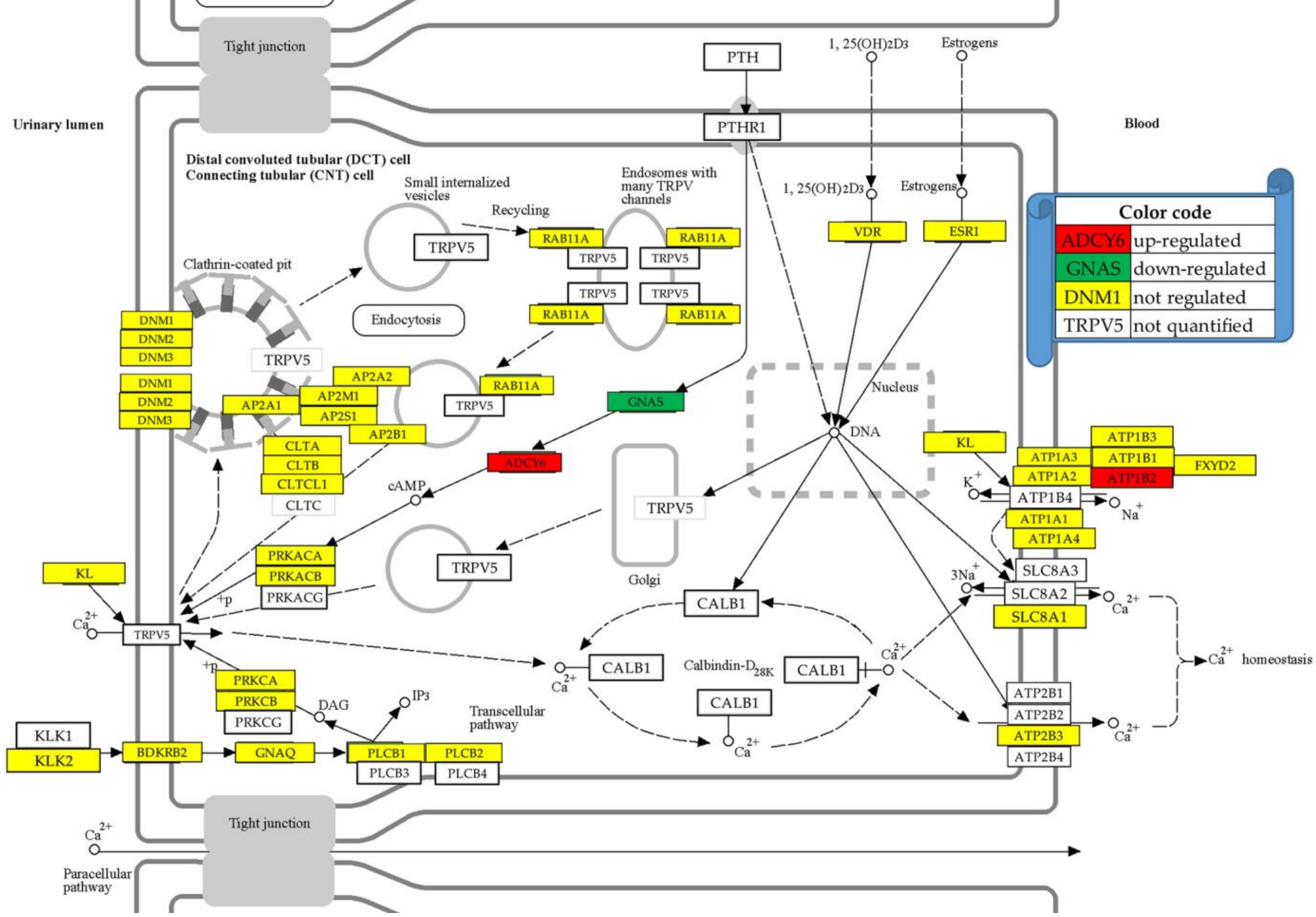

**(b) PTB**

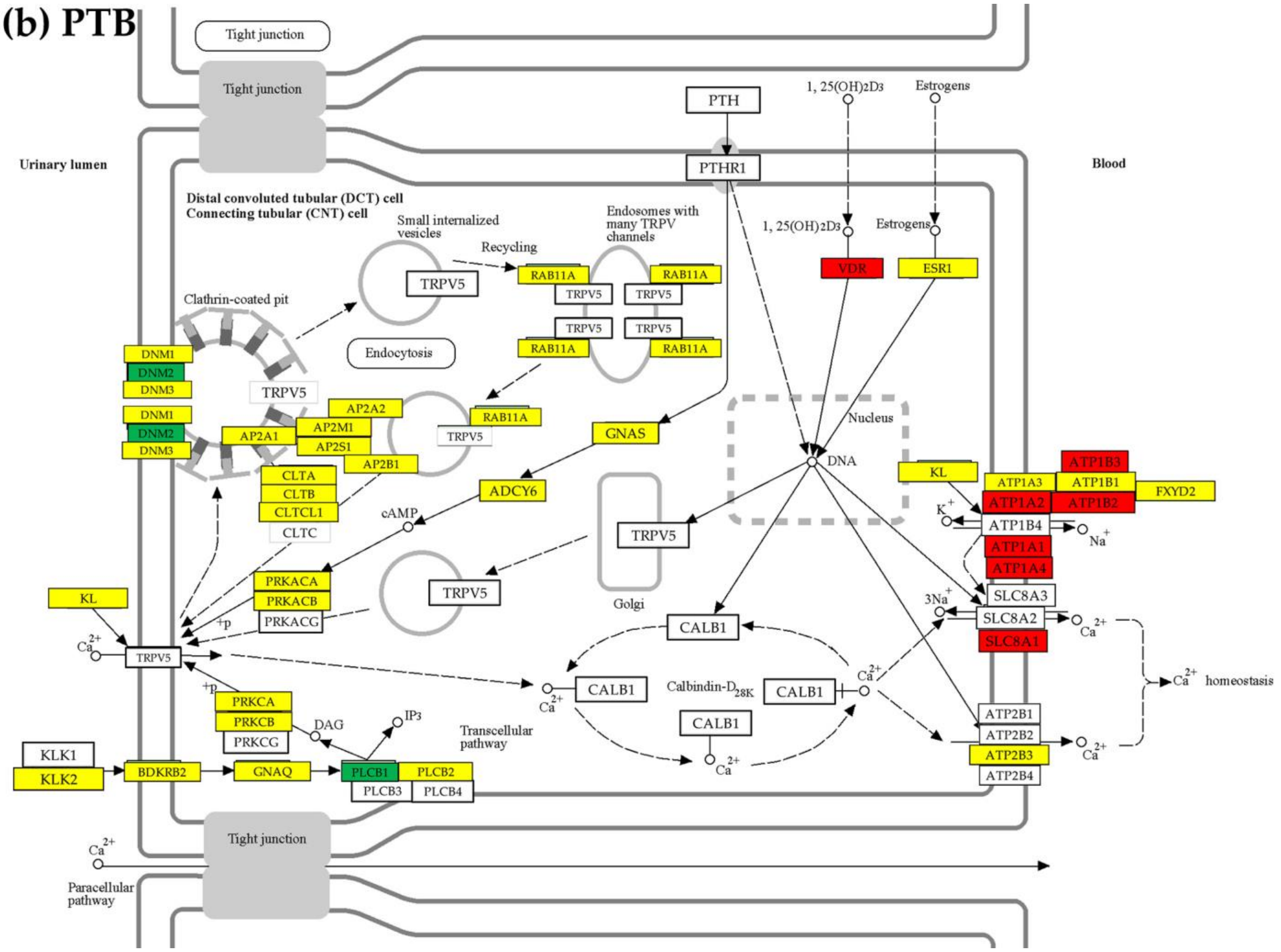

Tight junction
Tight junction
Urinary lumen
Blood
PTH
PTHR1
1, 25(OH)2D3
Estrogens
Distal convoluted tubular (DCT) cell
Connecting tubular (CNT) cell
Small internalized vesicles
Recycling
Endosomes with many TRPV channels
Clathrin-coated pit
Endocytosis
TRPV5
RAB11A
VDR
ESR1
DNM1
DNM2
DNM3
AP2A1
AP2A2
AP2M1
AP2S1
AP2B1
CLTA
CLTB
CLTCL1
CLTC
GNAS
ADCY6
cAMP
Nucleus
DNA
KL
ATP1B3
ATP1A3
ATP1B1
ATP1A2
ATP1B2
FXYD2
ATP1B4
ATP1A1
ATP1A4
SLC8A3
SLC8A2
SLC8A1
Golgi
PRKACA
PRKACB
PRKACG
CALB1
Calbindin-D28K
Ca2+ homeostasis
PRKCA
PRKCB
PRKCG
DAG
IP3
Transcellular pathway
ATP2B1
ATP2B2
ATP2B3
ATP2B4
KLK1
KLK2
BDKRB2
GNAQ
PLCB1
PLCB2
PLCB3
PLCB4
Paracellular pathway

**(c) CWM**

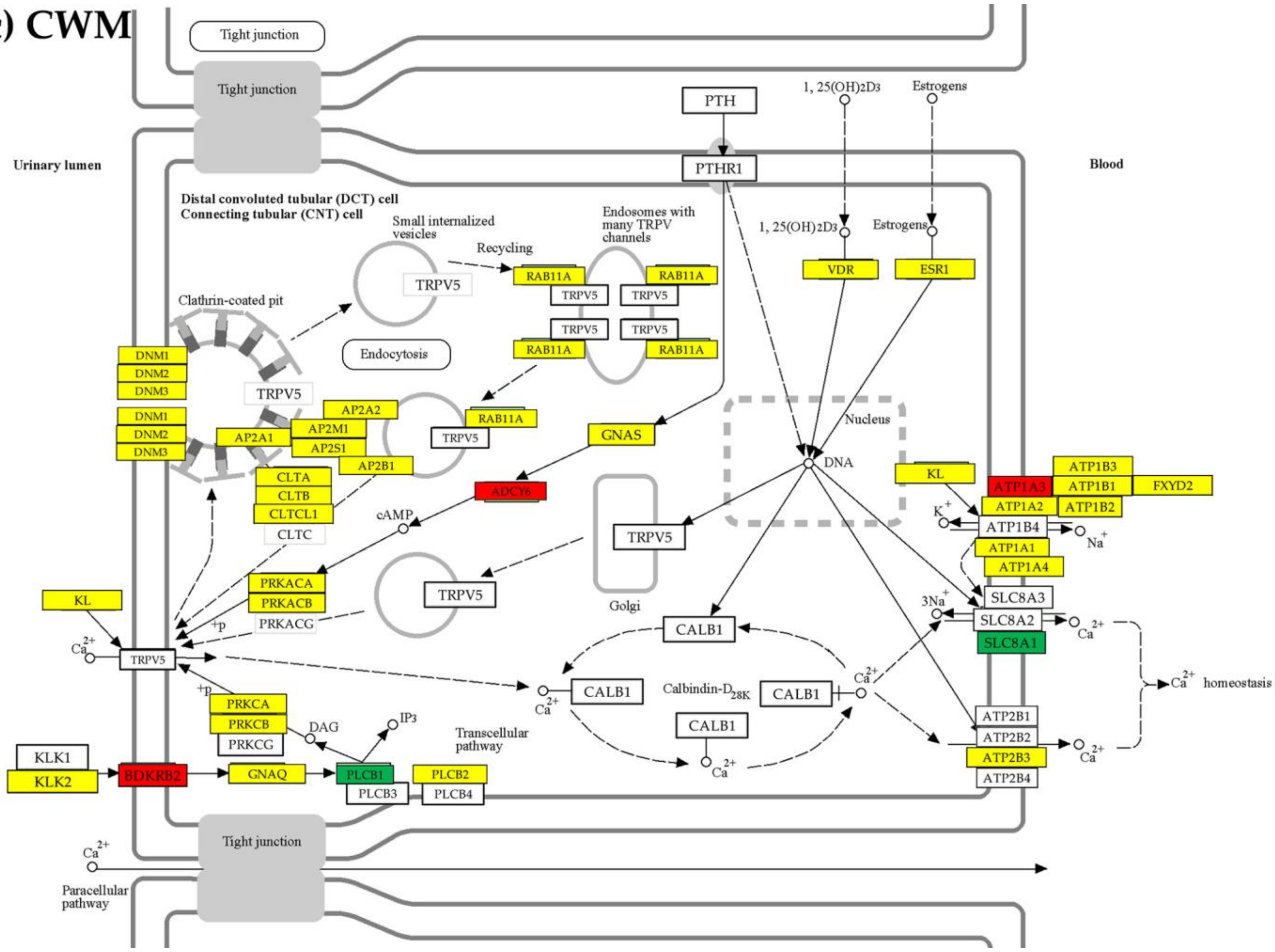

Tight junction
Tight junction
Urinary lumen
PTH
1, 25(OH)2D3
Estrogens
PTHR1
Blood
Distal convoluted tubular (DCT) cell
Connecting tubular (CNT) cell
Small internalized vesicles
Endosomes with many TRPV channels
Recycling
1, 25(OH)2D3
Estrogens
VDR
ESR1
RAB11A
RAB11A
TRPV5
TRPV5
TRPV5
Clathrin-coated pit
TRPV5
TRPV5
RAB11A
RAB11A
DNM1
DNM2
DNM3
Endocytosis
TRPV5
DNM1
DNM2
DNM3
AP2A1
AP2A2
AP2M1
AP2S1
AP2B1
RAB11A
TRPV5
GNAS
Nucleus
DNA
KL
ATP1B3
ATP1A3
ATP1B1
FXYD2
ATP1A2
ATP1B2
ATP1B4
ATP1A1
ATP1A4
CLTA
CLTB
CLTCL1
CLTC
ADCY6
cAMP
TRPV5
TRPV5
Golgi
PRKACA
PRKACB
PRKACG
+p
KL
SLC8A3
SLC8A2
SLC8A1
TRPV5
CALB1
CALB1
Calbindin-D28K
CALB1
CALB1
homeostasis
PRKCA
PRKCB
PRKCG
+p
DAG
IP3
Transcellular pathway
ATP2B1
ATP2B2
ATP2B3
ATP2B4
KLK1
KLK2
BDKRB2
GNAQ
PLCB1
PLCB2
PLCB3
PLCB4
Tight junction
Paracellular pathway

**Supplementary Figure S4:** Regulated genes in the KEGG-constructed functional pathway hsa04964 "Proximal tubule bicarbonate reclamation in the three profiled cancer nodules". **(a)** PTA nodule. **(b)** PTB nodule. **(c)** CWM nodule. **Regulated genes:** *ATP1A1/2/3/4* (ATPase, Na+/K+ transporting, alpha 1/2/3/4 polypeptide), *ATP1B2/3* (ATPase, Na+/K+ transporting, beta 2/3 polypeptide), *CA2* (carbonic anhydrase II), *PCK1* (phosphoenolpyruvate carboxykinase 1 (solu- 49 ble)), *SLC4A4* (solute carrier family 4 (sodium bicarbonate cotransporter), member 4).

**(a) PTA**

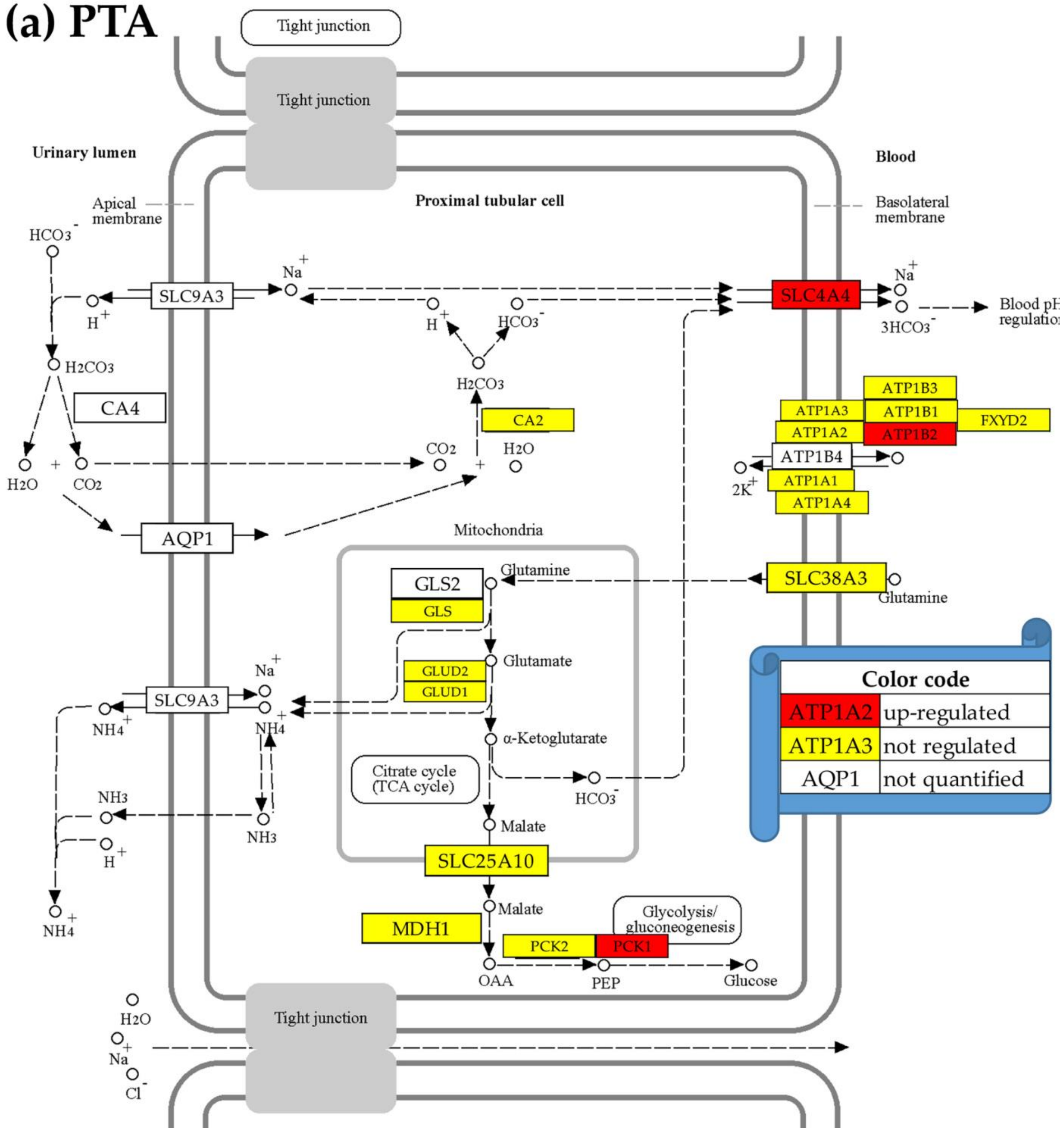

**(b) PTB**

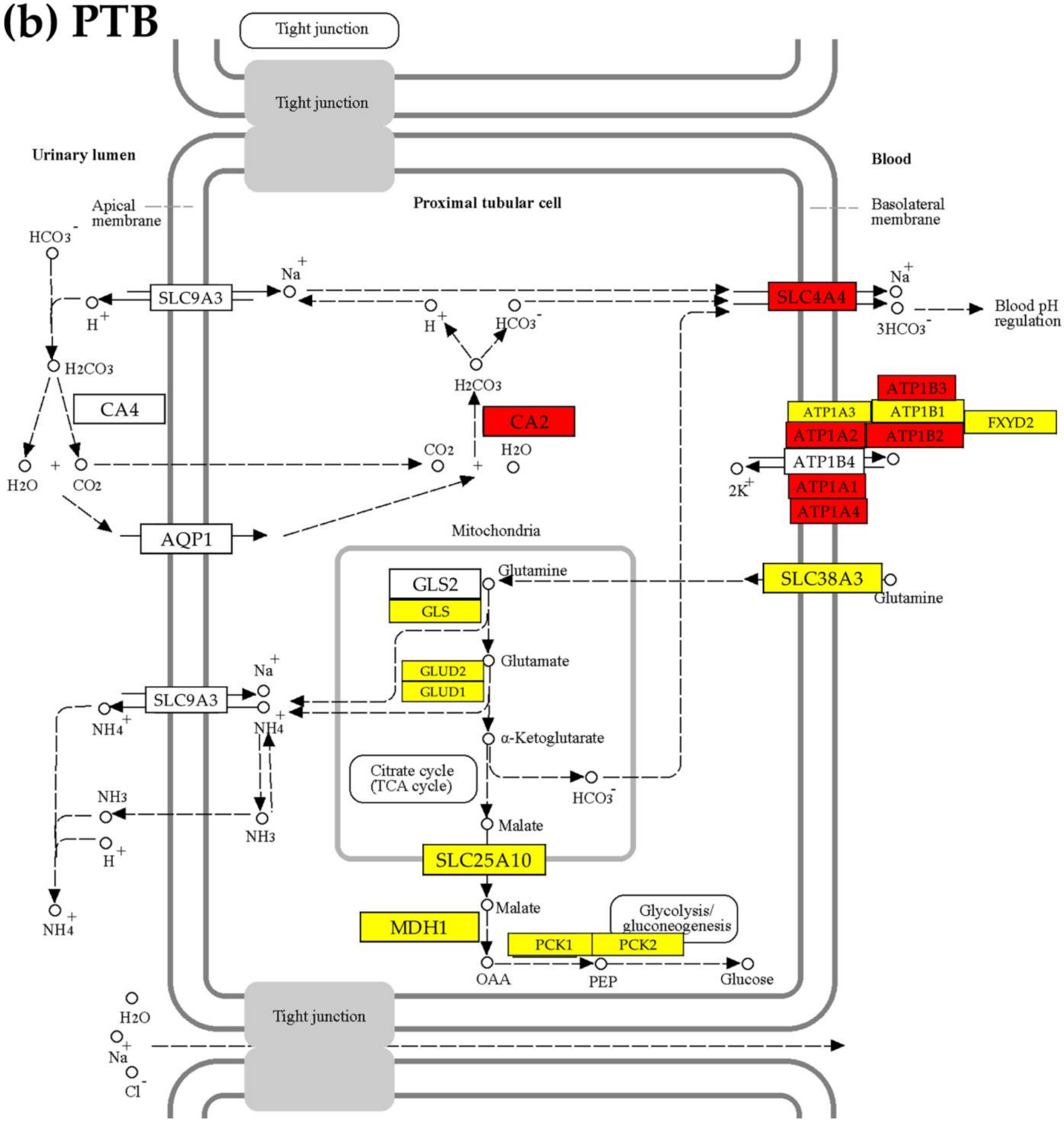
Tight junction
Tight junction
Urinary lumen
Blood
Apical membrane
Proximal tubular cell
Basolateral membrane
HCO3-
SLC9A3
Na+
H+
H2CO3
CA4
H2O
CO2
AQP1
H+
HCO3-
H2CO3
CA2
CO2
H2O
SLC4A4
Na+
3HCO3-
Blood pH regulation
ATP1B3
ATP1A3
ATP1B1
FXYD2
ATP1A2
ATP1B2
ATP1B4
2K+
ATP1A1
ATP1A4
Mitochondria
GLS2
GLS
Glutamine
SLC38A3
Glutamine
Glutamate
GLUD2
GLUD1
SLC9A3
Na+
NH4+
NH4+
α-Ketoglutarate
Citrate cycle (TCA cycle)
HCO3-
NH3
NH3
H+
NH4+
Malate
SLC25A10
Malate
MDH1
Glycolysis/ gluconeogenesis
PCK1
PCK2
OAA
PEP
Glucose
Tight junction
H2O
Na+
Cl-

**(c) CWM**

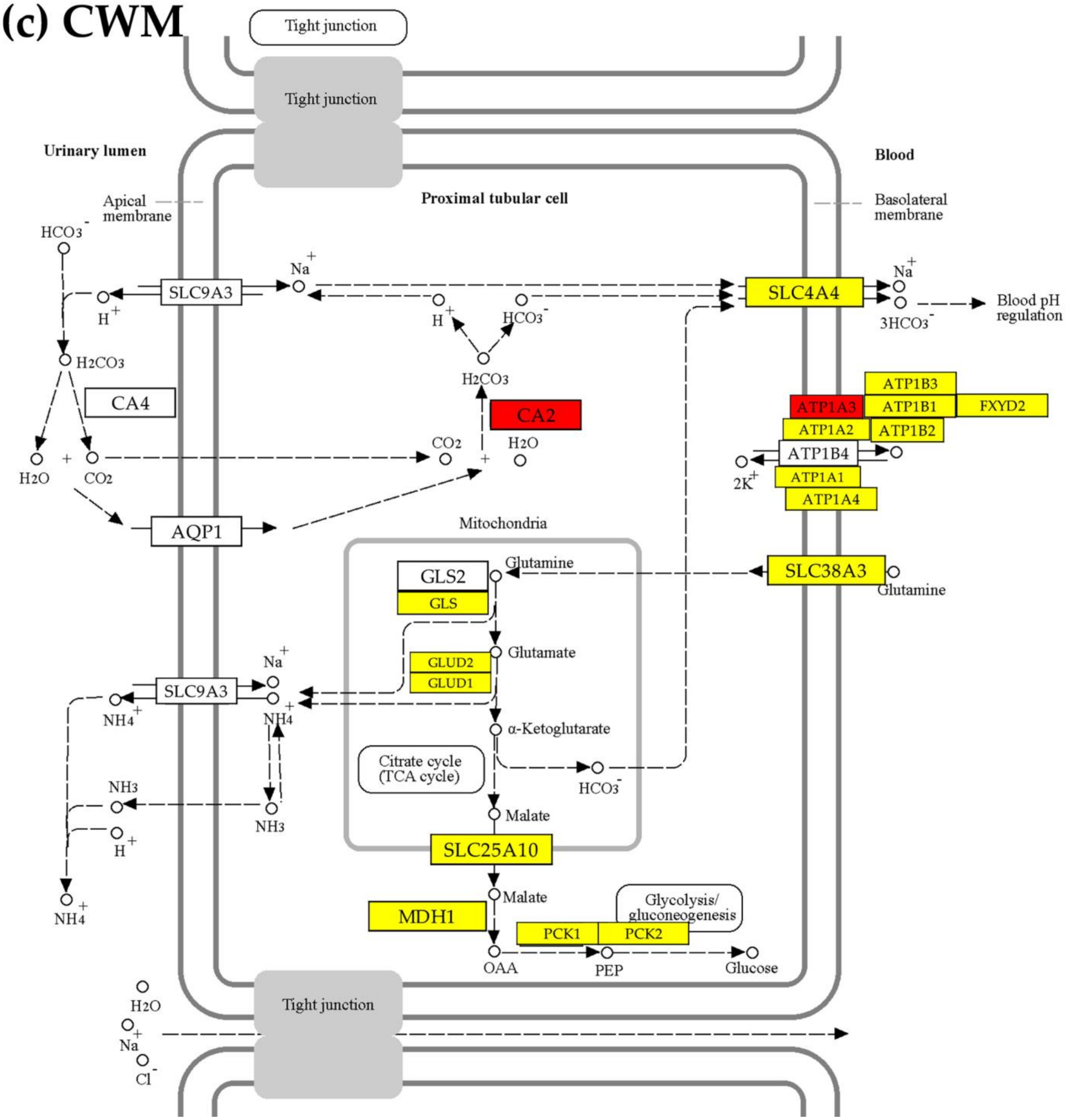

Tight junction
Tight junction
Urinary lumen
Blood
Apical membrane
Proximal tubular cell
Basolateral membrane
HCO3-
SLC9A3
Na+
SLC4A4
Na+
H+
H+
HCO3-
3HCO3-
Blood pH regulation
H2CO3
H2CO3
CA4
CA2
ATP1B3
ATP1A3
ATP1B1
FXYD2
ATP1A2
ATP1B2
CO2
H2O
ATP1B4
H2O
CO2
2K+
ATP1A1
ATP1A4
AQP1
Mitochondria
GLS2
Glutamine
SLC38A3
Glutamine
GLS
Glutamate
Na+
GLUD2
GLUD1
SLC9A3
NH4+
NH4+
α-Ketoglutarate
Citrate cycle (TCA cycle)
HCO3-
NH3
NH3
H+
Malate
SLC25A10
Malate
NH4+
MDH1
Glycolysis/ gluconeogenesis
PCK1
PCK2
OAA
PEP
Glucose
H2O
Tight junction
Na+
Cl-
